# A senescence stress secretome is a hallmark of therapy-related myeloid neoplasm stromal tissue occurring soon after cytotoxic exposure

**DOI:** 10.1038/s41375-022-01686-y

**Published:** 2022-08-29

**Authors:** Monika M. Kutyna, Chung Hoow Kok, Yoon Lim, Elizabeth Ngoc Hoa Tran, David Campbell, Sharon Paton, Chloe Thompson-Peach, Kelly Lim, Dimitrios Cakouros, Agnes Arthur, Timothy Hughes, Sharad Kumar, Daniel Thomas, Stan Gronthos, Devendra K. Hiwase

**Affiliations:** 1 Adelaide Medical School, Faculty of Health and Medical Sciences, University of Adelaide, Adelaide, SA Australia; 2 Precision Medicine Theme, South Australian Health and Medical Research Institute, Adelaide, SA Australia; 3 Centre for Cancer Biology, University of South Australia and SA Pathology, Adelaide, SA Australia; 4 Wakefield Orthopaedic Clinic, Calvary Wakefield Hospital, Adelaide, SA Australia; 5 Department of Haematology, Royal Adelaide Hospital, Adelaide, SA Australia

**Keywords:** Myelodysplastic syndrome, Cancer microenvironment

## Abstract

Therapy-related myeloid neoplasm (tMN) is considered a direct consequence of DNA damage in hematopoietic stem cells. Despite increasing recognition that altered stroma can also drive leukemogenesis, the functional biology of the tMN microenvironment remains unknown. We performed multiomic (transcriptome, DNA damage response, cytokine secretome and functional profiling) characterization of bone marrow stromal cells from tMN patients. Critically, we also compared (i) patients with myeloid neoplasm and another cancer but without cytotoxic exposure, (ii) typical primary myeloid neoplasm, and (iii) age-matched controls to decipher the microenvironmental changes induced by cytotoxics vs. neoplasia. Strikingly, tMN exhibited a profoundly senescent phenotype with induction of *CDKN1A* and β-Galactosidase, defective phenotype, and proliferation. Moreover, tMN stroma showed delayed DNA repair and defective adipogenesis. Despite their dormant state, tMN stromal cells were metabolically highly active with a switch toward glycolysis and secreted multiple pro-inflammatory cytokines indicative of a senescent-secretory phenotype that inhibited adipogenesis. Critically, senolytics not only eliminated dormant cells, but also restored adipogenesis. Finally, sequential patient sampling showed senescence phenotypes are induced within months of cytotoxic exposure, well prior to the onset of secondary cancer. Our data underscores a role of senescence in the pathogenesis of tMN and provide a valuable resource for future therapeutics.

## Introduction

Therapy-related myeloid neoplasms (tMN) are associated with dismal outcomes in otherwise long-term cancer survivors and are considered to be a direct consequence of DNA damage induced in hematopoietic stem cells (HSC) by chemotherapy and/or radiotherapy (cytotoxic therapies; CT) [1]. With an aging population and increasing long-term survival of cancer patients worldwide, the incidence of tMN is rising, currently accounting for 15–20% of myelodysplastic syndromes (MDS) and acute myeloid leukemia (AML) [1].

tMN is considered to be HSC-autonomous disorder, in which initiation and progression are driven by stem cell-intrinsic genetic events induced by CT [2, 3]. Recent data however indicate that tMN is likely to be a multi-factorial process, with an underlying germline predisposition syndrome contributing to pathogenesis in at least 15–20% of cases [4, 5]. Furthermore, work using murine genetic models have shown that genetic changes in the microenvironment play a critical role in initiation and evolution of myeloid neoplasia (MN), including reduced function of genes such as *RAR-γ*, *Rb*, *Mib1*, *IκBα*, *Sipa1*, *Dicer1* and concordant loss of the *Egr1*, *Apc*, and *Trp53* in non-hematopoietic cells [6–11]. Rare but notable cases of donor cell derived leukemia in clinical practice arising in patients receiving allogeneic stem cell transplantation aptly illustrates the concept of a microenvironment-driven disease [12].

A number of reports investigating stroma in MDS and AML have documented differences in osteogenic [13–16] and adipogenic potential [13, 15, 17], with diminished stem cell supportive capacity [13–15, 17, 18] but without comparison to tMN. CT causes short-term damage to the marrow (BM) microenvironment [19–21] but whether there is long-term damage and a pathogenic role for a perturbed stroma in the aetiology of tMN is unknown. Insights into stromal-derived factors that contribute to tMN are critical for preventing or delaying disease onset and targeting the interaction between HSC and microenvironment.

Here, we performed a comprehensive multi-omic characterization of BMSC from patients with tMN and found evidence of a profound pro-inflammatory senescence program and a selective defect in adipogenesis which was reversed with senolytic therapy, with implications for future strategies to delay or prevent the onset of tMN.

## Materials and methods

*Please refer to* *Supplementary Information**for detailed “Materials and Methods” description*.

### Patients and control samples

Diagnostic BM samples from MN patients, and age-matched controls at the time of hip replacement were collected after informed consent. In a subset of tMN patient sequential samples were collected. The study was approved by the Ethics Committees of participating institutions (Central Adelaide Local Health Network- HREC reference number: HREC/15/RAH/496, CALHN reference number: R20151123 and Calvary Health Care Adelaide- 19-CHREC-E004) and performed in accordance with the Declaration of Helsinki. Informed consent was obtained from all subjects.

### Isolation, expansion, and characterization of BMSC

BMSC were isolated and expanded from mononuclear cells according to criteria of the International Society for Cellular Therapy including immunophenotype characteristics [22], cytoskeleton [18], proliferative potential, osteoblast and adipocyte differentiation potential [23].

### Long-term culture-initiating cells assay

Stem/progenitor supportive capacity was assessed by co-culturing HSC on BMSC feeder-layer in long-term culture initiating assay for 5 weeks followed by clonogenic assay for additional 2 weeks [24].

### Analysis of apoptosis, DNA damage and cellular senescence

Apoptosis and cellular senescence were assessed at third passage using Annexin-V-PE Kit (BD Biosciences) and β-Galactosidase (Cell Signaling Technology) respectively. DNA damage repair (DDR) potential was assessed by phosphorylation of γH2AX (Merck) and alkaline comet assay (Abcam). While reactive oxygen species (ROS) production was determined by measuring the formation of fluorescent dichloro fluorescein at an Excitation 485 and Emission 535 nm (Abcam).

### Western immunoblotting

Response to in vitro DNA damaging radiotherapy was assessed in tMN and Healthy BMSC by assessing phosphorylation of ataxia telangiectasia mutated (ATM), chekpoint-2 (CHK2), p53 and p21 levels at 0.5, 1, 3, 5, 8, and 24 h following 20 Gy irradiation.

### Transcriptome analysis

Transcriptome sequencing libraries were prepared with Illumina TruSeq Total RNA protocol (Illumina, San Diego) for 150 bp paired-end sequencing on a NextSeq500 instrument. Gene-set enrichment analysis was performed using Broad Institute software version 4.0 and Molecular Signature Database (MSigDB) version 7.2. Genes differentially were validated in expanded cohort by quantitative real-time PCR.

### Cytokines/chemokines levels

Levels of 38 cytokines were measured with HCYTOMAG-60K MILLIPLEX MAP Human Cytokines/Chemokines magnetic bead panel.

### Real-time ATP rate assay

ATP production was measured using the Seahorse XF ATP Real-Time rate assay (Agilent Technologies).

### Statistical analysis

Data analysis and graphs were generated using GraphPad prism 8 software. Statistical analysis was performed using Mann–Whitney, Student’s *t* test, or Two-way ANOVA to determine difference between groups. Normality test was performed using GraphPad prism 8 software. *P* < 0.05 was considered statistically significant. Details regarding statistical methods for transcriptome sequencing, quantitative real-time PCR, and cytokines/chemokines heatmap are explained in supplementary section.

## Results

**Fig. 1:**
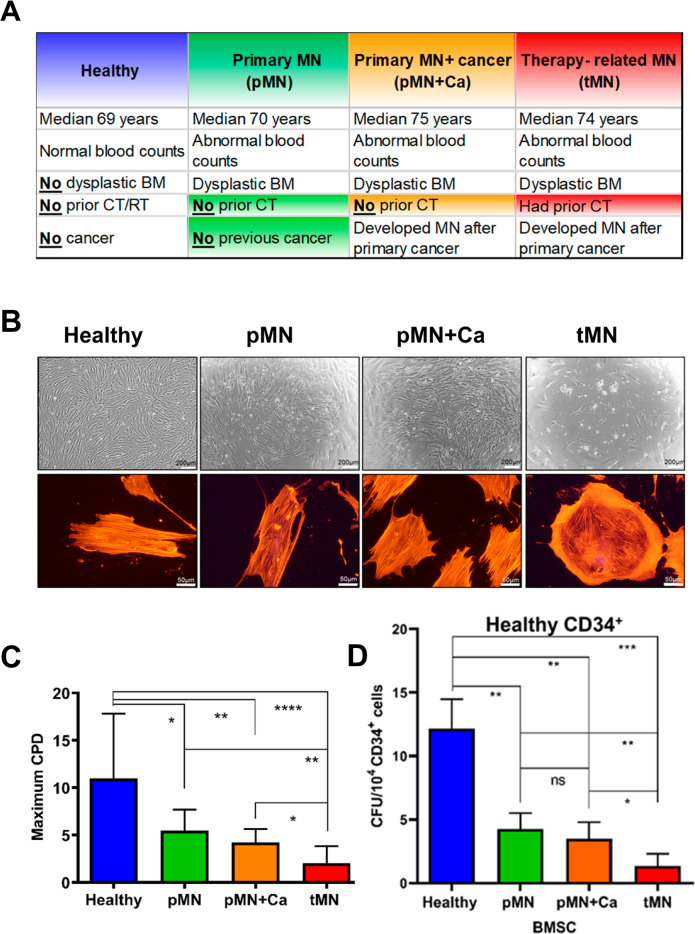
Aberrant morphology and growth kinetics of bone marrow mesenchymal stromal cells (BMSC) isolated from therapy-related myeloid neoplasms (tMN) patients. **A** Crucial differences between carefully selected four cohorts (Healthy *n* = 17; pMN *n* = 7; pMN+Ca; *n* = 10; tMN *n* = 18); **B** representative micrographs of BMSC morphology assessed by light microscopy (upper panel) and cytoskeleton by TRITC-Phalloidin stain (lower panel). Scale bars indicate 200 μm and 50 μm respectively; **C** maximum cumulative population doublings (CPD); **D** in vitro HSC supportive capacity of BMSC assessed by co-culturing Healthy (blue bar; *n* = 2), pMN (green bar; *n* = 2), pMN+Ca (orange bar; *n* = 2) and tMN (red bar; *n* = 6) BMSC with Healthy CD34^+^ cells for 7 weeks followed by clonogenic assays. All bars indicate mean, and all error bars indicate SD. Mann–Whitney test was used to detect statistically significant differences between cohorts. Asterisks display *P* values *P < 0.05, **P < 0.01, ***P < 0.001, **** *P* < 0.0001.

Our preliminary observations of a striking defect in the proliferative capacity of BMSC from tMN patients (*n* = 20) compared to MDS/AML (*n* = 26), as well as to healthy controls (*n* = 13) (Fig. S1A–C) led to further comprehensive analysis. To decipher effects specifically induced by cytotoxic therapies rather than age, we performed multi-omic profiling (RNA-sequencing, cytokines levels, proliferation potential, multilineage differentiation and DNA damage) in BMSC from four carefully selected cohorts (Fig. 1A): (i) tMN, in which a MN occurred in a cancer survivor following CT (*n* = 18); (ii) MN developing in unrelated cancer survivors with *no prior CT* (a diagnosis of primary MN and another cancer; pMN+Ca) (*n* = 10). For example, this group included MDS/AML occurring in patients managed with surgical resection of prostate cancer but no CT; (iii) primary MN without preceding cancer and/or CT (pMN) (*n* = 7) and (iv) age-matched healthy controls (Healthy) (*n* = 17). The critical difference between tMN and pMN+Ca is prior to CT in tMN but not in pMN+Ca. Demographic and disease parameters of each cohort are detailed in Table S1. Cytotoxic therapy details used for treating primary cancer in tMN patients are listed in Table S2.

Out of 18 tMN patients, 78% had documented previous chemotherapy with or without radiotherapy, whereas 22% received only radiotherapy. There was no significant difference in median age of Healthy (69 years, range 43–92) and MN patients (73 years, range 58–87) or expression of stromal markers (Fig. S2A).

### tMN stromal cells display abnormal morphology and defective growth kinetics

The most striking difference apparent upon culturing was in morphology. tMN BMSC were significantly larger (27 ± 10 mm) than BMSC from pMN (16.8 ± 3 mm, *P* < 0.001), pMN+Ca (16.9 ± 2 mm, *P* = 0.013) and Healthy (14.5 ± 4 mm, *P* < 0.001) (Fig. S2B) and lost the typical spindle shape morphology (Fig. 1B), resembling a flattened senescent cell [25]. BMSC from pMN and pMN+Ca were disorganized and varied in size compared to controls but overall maintained a fibroblast-like morphology (Fig. 1B).

Overall, for all MN groups including tMN, BMSC could be cultured for significantly fewer number of passages than Healthy (6 ± 2 vs. 11 ± 4 passages, *P* < 0.001) (Fig. S2C). This was reflected by a decrease in maximum cumulative population doublings (CPD) (3.2 ± 2 vs. 11 ± 7 doublings, *P* < 0.001, Fig. 1C) and the number of fibroblast colony-forming units compared with Healthy (1.2 ± 1 vs. 4.5 ± 3 colonies, *P* < 0.001) (Fig. S2D). However, tMN appeared to have the greatest defect, with 28% of tMN BMSC failed to reach the first passage and only 45% could be cultured beyond passage 3, significantly lower than pMN and pMN+Ca groups (*P* < 0.001, *P* = 0.005) (Fig. S2E). CPD was significantly lower in tMN (2 ± 2) compared to pMN (5.5 ± 2) and pMN+Ca (3.7 ± 2) (*P* = 0.001 and *P* = 0.029 respectively) (Fig. 1C). Furthermore, tMN BMSC were associated with longer doubling times (27 ± 35 vs. 12 ± 7 vs. 14 ± 12 days respectively) (Fig. S2F) and decreased colony numbers compared to pMN and pMN+Ca (0.38 ± 0.8, 1.9 ± 0.4 and 2.1 ± 0.6 per 10^4^ cells respectively, *P* < 0.001) (Fig. S2D).

Consistent with the observed aberrant morphology and growth defects, tMN stroma were unable to support the long-term culture of normal healthy CD34^+^ in long-term culture initiating assays (Fig. 1D). tMN BMSC exhibited a 12-fold decreased capacity to support Healthy CD34^+^ compared with Healthy BMSC (1 ± 1 vs. 12 ± 2 colonies; *P* < 0.001) and 3–4 folds lower than pMN (*P* = 0.001) and pMN+Ca (*P* = 0.014) (Fig. 1D). Consistently, functional enrichment of transcriptome analysis showed depletion of hematopoietic support/regulation pathways in tMN BMSC compared to Healthy and other MN (Fig. S3A–B). Moreover, *CXCL12* expression, a key regulator of hematopoiesis was significantly lower in tMN BMSC compared to Healthy (*P* < 0.001) and pMN (*P* = 0.007) (Fig. S4A). Collectively, these findings demonstrate significantly impaired proliferative capacity of tMN BMSC compared to other MN groups without prior CT exposure.

### tMN stromal cells exhibit a profoundly senescent phenotype

**Fig. 2:**
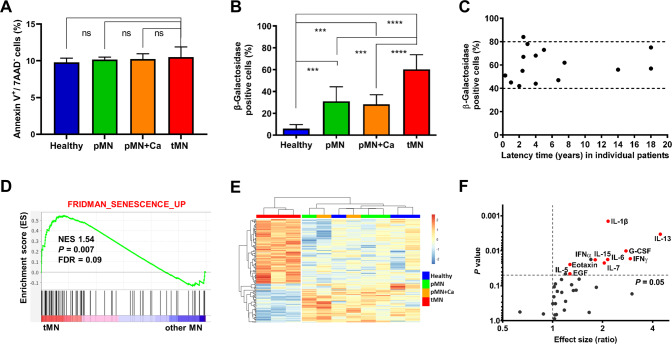
Profound senescent phenotype in tMN BMSC. **A** Apoptotic cells at passage 3 defined as Annexin V positive (Annexin V^+^) and 7AAD negative (7AAD^-^) cells (Healthy *n* = 4; pMN *n* = 4; pMN+Ca *n* = 4; tMN *n* = 4); **B** cellular senescence assessed by percentage of β-Galactosidase-stained cells at passage 3 (Healthy *n* = 17; pMN *n* = 7; pMN+Ca *n* = 10; tMN *n* = 18); **C** association between cellular senescence assessed by percentage of β-Galactosidase-stained cells at passage 3 and latency time (time from last dose of CT for primary cancer to tMN diagnosis) (tMN *n* = 18); **D** example of GSEA plot showing the specific enhancement of gene set associated with cellular senescence in tMN compared to other MN BMSC. The normalized enrichment score (NES), *P* values and false discovery rate (FDR) are given; **E** heatmap representing the gene expression changes of senescence-associated genes found to be significantly differently deregulated (FDR *P* < 0.1 and log2 FC > | 0.6 | ) in the BMSC from tMN as compared to Healthy and other MN BMSC. A total of 144 genes involved in the senescence (SeneQuest Database), were analyzed; **F** levels of 14 SASP-cytokines in tMN BMSC-conditioned media compared Healthy (Healthy *n* = 17; tMN *n* = 17). Cytokines in red are significantly higher and are SASP-related. All bars indicate mean, and all error bars indicate SD. Mann–Whitney test was used to detect statistically significant differences between cohorts. Asterisks display *P* values ***P < 0.001, ****P < 0.0001.

Although colony forming and proliferative capacity of tMN BMSC was significantly compromised, apoptosis rate was not significantly different as compared to Healthy and other typical MN BMSC (pMN and pMN+Ca) (Fig. 2A). Moreover, morphological indicators of senescence as measured by β-Galactosidase staining were markedly elevated in tMN (60 ± 13%) compared to pMN (31 ± 13%), pMN+Ca (28.29 ± 9%) and Healthy control (6 ± 4%) (*P* < 0.001) (Fig. 2B) with a corresponding increase in *CDKN1A* (p21) and decrease in *FOS* gene expression in tMN (Fig. S4B–C). Cellular senescence was higher in all MN groups compared to Healthy (45 ± 19% vs. 6 ± 4%, *P* < 0.001) (Fig. 2B). The level of senescence in tMN BMSC was independent of latency period, the interval between completion of CT and tMN diagnosis. High levels of senescence were evident both in tMN BMSC with short (3–4 months) and tMN with long latency reaching up to two decades following CT (Fig. 2C).

Overall, transcriptomic analysis was consistent with a severe senescent profile in tMN compared to Healthy and other MN BMSC over multiple enrichment analysis sets (Fig. 2D and Fig. S3A–B). Moreover, substantial number of the 147 genes differentially expressed between tMN and other MN BMSC were reported to be associated with cellular senescence on SenQuest (*P* < 0.001) (Fig. S5A) and CellAge (*P* < 0.001) (Fig. S5B) senescence databases. Further analysis of senescence-associated genes revealed distinct signature of tMN BMSC as compared to all other control cohorts (Fig. 2E). RT-PCR validation was performed on an extended independent cohort of tMN (*n* = 4), pMN (*n* = 3), pMN+Ca (*n* = 2) and Healthy (*n* = 4) for *CDKN1A, FOS, UHRF2* and *IFITM1* (Fig. S4B–E). Moreover, expression of 43 genes strongly correlated with β-Galactosidase levels including senescence-associated genes (Fig. S5C). A cardinal feature of cellular senescence is senescence-associated secretory phenotype (SASP), secretion of pro-inflammatory cytokines, chemokines and proteases that may serve as a homeostatic signal of cellular damage [26, 27]. Indeed, multiple SASP-associated cytokines including IL-6, IFNγ, IL-7, IL-1β, IL-13, IL-15, and EGF were found to be significantly higher in conditioned media of tMN BMSC compared to Healthy (Fig. 2F). In agreement with previous reports [28–30], several SASP-associated cytokines levels were also higher in other MN (non-tMN), BMSC conditioned media compared to Healthy (Fig. S6).

### tMN stromal cells have defects in DNA damage repair

**Fig. 3:**
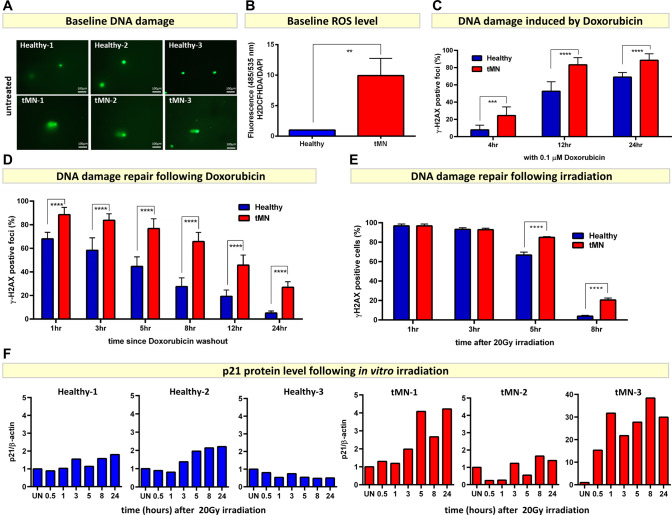
tMN stromal cells exhibit defective DNA damage repair potential. **A** Alkaline comet assay shows high level of baseline DNA damage in tMN (*n* = 3) compared to Healthy (*n* = 3) BMSC. The length of the comet tail reflects the number of DNA breaks; **B** high level of baseline DNA damage is probably driven by high level of ROS in tMN compared to Healthy BMSC; **C** significantly high number of γH2AX foci formation were evident after 4, 12, and 24 h of exposure to 0.1 *µ*M Doxorubicin, a commonly used chemotherapeutic drug; **D** defective DNA damage repair potential of tMN BMSC was evident by significantly high number of γH2AX foci at all time points following Doxorubicin drug-washout; **E** following in vitro radiation, number of γH2AX foci in Healthy BMSC decreased with only 4 ± 1% cells showing positivity by 8 h after irradiation. However, tMN BMSC showed significantly impaired repair with 21 ± 2% foci still positive at the same time point; **F** densitometry analysis of Western blots showing p21 protein expression normalized to β-actin in three tMN and Healthy BMSC analyzed. UN, untreated. All bars indicate mean, and all error bars indicate SD. Mann–Whitney test was used to detect statistically significant differences between two groups and Two-way ANOVA was used to determine differences among groups. Asterisks display *P* values **P < 0.01, **** *P* < 0.0001.

The level of high senescence observed in tMN BMSC could be due to a defect in the DNA damage repair (DDR) pathway [31], resulting in irreparable DNA damage after exposure to cytotoxic therapy. To assess this, we first examined baseline DNA damage in tMN and Healthy BMSC. BMSC derived from tMN exhibited higher baseline DNA damage, as shown by alkaline comet assay, compared to Healthy BMSC (Fig. 3A and Fig. S7A). Enhanced baseline DNA damage in tMN BMSC was probably driven by higher intracellular ROS level compared to Healthy BMSC (*P* = 0.001) (Fig. 3B). Secondly, tMN BMSC were highly sensitive to DNA damaging CT. High number of γH2AX foci formation were evident within 4 h (8 ± 5 vs. 24 ± 10%; *P* < 0.001) and peaking within 12 h of in vitro exposure to 0.1 µM Doxorubicin (53 ± 11 vs. 83 ± 8%; *P* < 0.001) (Fig. 3C). Similar trend was observed with 1 µM Doxorubicin (Fig. S7C). Finally, tMN BMSC displayed defective DDR capacity following in vitro exposure to chemotherapeutic drug and radiation. Following 24 h in vitro exposure and drug-washout, γH2AX foci were consistently significantly higher in tMN BMSC at all time points and persistent DNA damage was evident even after 24 h of removal of genotoxic insult, compared to Healthy BMSC (27 ± 5% vs. 5 ± 2%; *P* < 0.001) (Fig. 3D). Similarly, tMN BMSC showed significantly impaired DDR following in vitro radiation exposure. Following in vitro radiation, number of γH2AX foci in Healthy BMSC decreased rapidly over time with only 4 ± 1% cells showing positivity by 8 h after irradiation (Fig. 3E). However, tMN BMSC showed significantly impaired repair with 21 ± 2% foci still positive at the same time point (*P* < 0.001) (Fig. 3E and Fig. S8A). This was also significantly greater than pMN and pMN+Ca samples (5 ± 1%, *P* < 0.001), indicating significantly delayed DDR response in tMN stroma compared to both Healthy and typical MN (Fig. S8A–B) and was consistent with Gene Set Enrichment (GSEA) analysis for pathways associated with DDR (FDR < 0.1, *P* < 0.05) (Fig. S3A–B).

Delayed DNA repair of tMN BMSC is probably driven by delayed recruitment and activation of the early responders following DNA damage. In Healthy BMSC ATM and CHK2 were activated on by serine 1981 and threonine 68 respectively within 30 min of in vitro sublethal irradiation (Fig. S9A). While, in tMN BMSC, there was a significant delay of up to 3 h in early responders, with some interpatient variability (Fig. S9B). Delayed activation was associated with late recruitment of ATM/CHK2/p53 and its crucial transcriptional target p21, a key mediator of senescence (Fig. 3F and Fig. S9). Of note, none of the 13 BMSC tested had pathogenic *TP53* mutations and all samples showed evidence of intact *TP53* pathway.

### tMN stromal cells show a glycolytic metabolic state

**Fig. 4:**
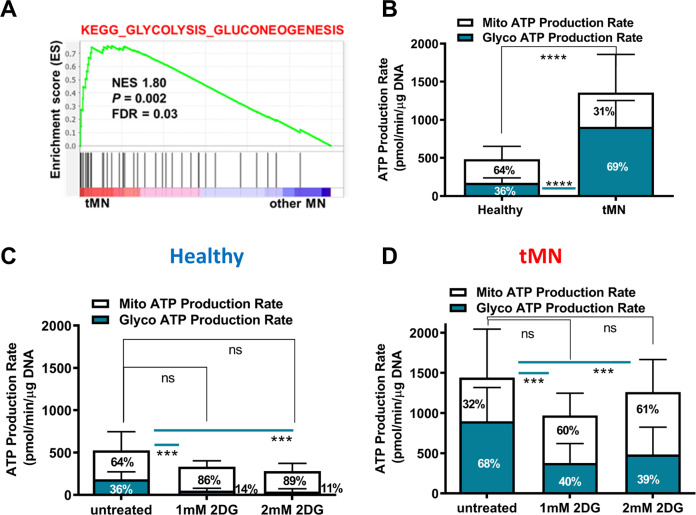
tMN BMSC display glycolytic metabolic state. **A** GSEA example of BMSC from tMN vs. other MN shows enrichment of metabolic pathways. The normalized enrichment score (NES), *P* values and false discovery rate (FDR) are given; **B** comparison of mitochondrial ATP (mitoATP) production rate and glycolytic ATP (glycoATP) production rate between Healthy (*n* = 3) and tMN BMSC (*n* = 5). Data were expressed relative to the total DNA. Effect of glycolysis inhibitor 2DG on mitochondrial and glycolytic production rate in **C** Healthy BMSC (*n* = 3) and **D** tMN BMSC (*n* = 3). All bars indicate mean, and all error bars indicate SD. Mann–Whitney test was used to detect statistically significant differences between cohorts. Asterisks display *P* values **P < 0.01, ****P < 0.001.

While the tMN stroma was profoundly dormant, it was certainly not inactive metabolically. Our transcriptome analysis showed enrichment of multiple glycolytic gene pathways in tMN BMSC compared to controls (FDR < 0.1, *P* < 0.05) (Fig. 4A and Fig. S3A–B). Moreover, bioenergetic profiling showed that total ATP production was significantly higher in tMN compared to Healthy BMSC (679 ± 482.6 pmol/min/µg DNA vs. 241.6 ± 144.5 pmol/min/µg DNA, *P* < 0.001) (Fig. 4B). Notably, the majority of ATP in tMN BMSC was generated by glycolysis (69% vs. 36%; *P* < 0.001), contrasting with Healthy in which ATP was predominantly generated by mitochondrial oxidative phosphorylation (OXPHOS) (64% vs. 31%) (Fig. 4B and Fig. S10A–C). Moreover, the extracellular acidification rate (ECAR), an indicator of glycolysis was increased in tMN compared to Healthy BMSC (Fig. S10B). We therefore tested whether glycolytic ATP production was hardwired in tMN stroma or could be switched to OXPHOS. As expected, glycolysis inhibitor, 2-Deoxy-D-glucose (2DG) significantly reduced glycolysis (*P* < 0.001) and ECAR in Healthy and tMN BMSC, but without reducing total ATP significantly (Fig. 4C–D), indicating a retained capacity to upregulate OXPHOS (Fig. S10D–I). Oxygen consumption rate (OCR) was similar in majority of Healthy and tMN BMSC, although interpatient variability was evident in tMN BMSC (Fig. S10C). In addition, glycolysis inhibitor 2DG did not affect OCR in both Healthy and tMN BMSC (Figs. S10F, S10I). Together, the data suggest that tMN senescent cells despite a low proliferation rate, exhibit a perturbed active glycolytic metabolism in normoxia, resembling a Warburg metabolic shift.

### tMN stromal cells show defective adipogenesis and secrete adipogenic-inhibitory cytokines

**Fig. 5:**
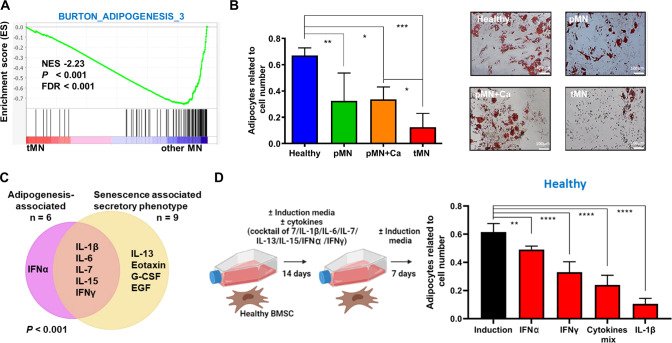
Defective adipogenesis of tMN BMSC. **A** GSEA plot showing the specific deregulation of gene sets associated with adipogenesis in BMSC from tMN and other MN. NES, FDR and *P* values are given; **B** quantification of Nile red-stained lipid droplets, relative to total number of DAPI stained cells. BMSC were cultured under adipogenic inductive conditions for 4 weeks (Healthy *n* = 8; pMN *n* = 5; pMN+Ca *n* = 3; tMN *n* = 11). Representative micrographs of Oil red stained lipids. Scale bars indicate 100 μm; **C** Venn diagram representing significantly different cytokines related to SASP and adipogenesis in tMN BMSC compared to Healthy, as well as those cytokines which are in common in SASP and adipogenesis-associated groups; **D** schematic of the Healthy BMSC co-culture experiments with various cytokines. Healthy BMSC were cultured in adipogenesis-induced media with either seven cytokines cocktail, or individual cytokines such as IL-1β, IL-6, IL-7, IL-13, IL-15, IFN2A or IFNγ. Following 14 days, Healthy BMSC were cultured without cytokines for 7 days and adipogenic differentiation was investigated. The number of adipocytes was enumerated by counting Nile Red-labelled cells and DAPI-labelled cell nuclei. The results from three Healthy BMSC are shown (*n* = 3). All bars indicate mean, and all error bars indicate SD. Mann–Whitney test was used to detect statistically significant differences between cohorts. Asterisks display *P* values *P < 0.05, **P < 0.01, ***P < 0.001, ****P < 0.0001.

BMSC have several possible cell fates. Adipogenesis in particular depends on a robust metabolic reprogramming and our transcriptome data suggest that genes involved in adipogenesis were downregulated in tMN (FDR < 0.1, *P* < 0.05) (Fig. 5A and Fig. S3A–B). Consistently, BMSC derived from tMN displayed reduced expression of genes critically involved in the early and late adipogenic differentiation, namely *PPARγ* and Adipsin (Fig. S11).

To assess whether tMN stroma, despite glycolytic bias, was able to undergo adipogenic differentiation, we performed adipocyte differentiation assays. Unexpectedly, we noted that quantification of lipid-laden Nile Red marked adipocytes was significantly reduced compared to pMN+Ca (*P* = 0.024) and Healthy (*P* < 0.001) (Fig. 5B). These results were validated by staining with Oil Red (Fig. 5B).

To understand the mechanism of defective adipogenesis in tMN, we first focused on known inhibitors of adipogenesis from our cytokine bead array data. We found secretion of the adipocyte-inhibitory cytokines IL-1β, IL-15, IL-6, IFNα and IFNγ were significantly higher by tMN BMSC compared to Healthy (Fig. 2F) and noted a significant overlap between adipogenesis-inhibitory cytokines and pro-inflammatory cytokines known to be cardinal SASP cytokines (*P* < 0.001) [26, 27] (Fig. 5C). To confirm this experimentally, treatment of Healthy BMSC with IL-1β, IFNγ, IFNα or a cocktail of seven-cytokines (IL-1β, IL-13, IL-15, IL-6, IFNα and IFNγ) profoundly inhibited adipogenesis of in vitro (*P* < 0.003) (Fig. 5D and Fig. S12), demonstrating a potential causative role of senescence-secreted cytokines in inhibiting adipogenesis.

**Fig. 6:**
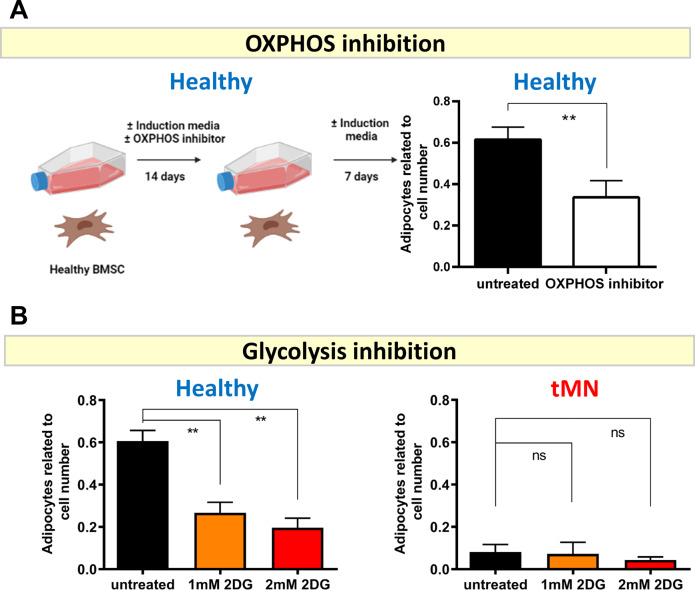
Inhibition of glycolysis does not restore defective adipogenesis in tMN BMSC. **A** Schematic showing that Healthy BMSC were cultured in adipogenesis-induction media with or without OXPHOS inhibitor (IACS-010759) for 14 days followed by 7 days without the inhibitor and adipogenesis was assessed after total of 21 days culture. The number of adipocytes was enumerated by counting Nile Red-labelled cells and DAPI-labelled cell nuclei. The bar represents average of results from three Healthy BMSC; **B** Healthy (*n* = 3) and tMN BMSC (*n* = 3) were cultured under adipogenic induction conditions for 4 weeks either with or without 1 mM and 2 mM of 2DG, a glycolysis inhibitor, and number of adipocytes were evaluated by counting Nile Red-labelled cells and DAPI-labelled cell nuclei. All bars indicate mean, and all error bars indicate SD. Scale bars indicate 100 µm. Mann–Whitney test was used to detect statistically significant differences between cohorts. Asterisks display *P* values **P < 0.05.

Secondly, we tested experimentally whether the extreme bioenergetic phenotype observed in tMN may also contribute to the defect in adipogenesis. We noted that blockade of either mitochondrial OXPHOS (Fig. 6A and Fig. S13A) or glycolysis (Fig. 6B and Fig. S13B) in Healthy BMSC led to significantly reduced adipogenesis (*P* < 0.001) consistent with the metabolic requirement of adipogenesis to store glucose-derived de novo fatty acids and also perform mitochondria fatty acid β-oxidation. Not surprisingly, switching to OXPHOS did not restore adipogenesis (*P* = 0.730) (Fig. 6B and Fig. S13B) in tMN BMSC. Collectively these results suggest tMN stroma is profoundly senescent but metabolically active, with a defective adipogenic phenotype likely linked to the production of adipocyte-inhibitory cytokines.

### tMN stromal cells have increased osteogenic differentiation potential

BMSC osteogenic differentiation potential, assessed by mineralization matrix formation, was impaired in pMN compared to Healthy BMSC (14.8 ± 23.7 vs. 53 ± 34.3 Ca^2+^/µg DNA, *P* = 0.029). In contrast to this, bone mineralization matrix capacity was significantly higher in tMN BMSC (182.7 ± 190.3 Ca^2+^/µg DNA) compared to Healthy controls and pMN (*P* = 0.013). This was further confirmed by Alizarin Red-stained mineralized deposits (Fig. S14A–B). Thus, in contrast to pMN, tMN BMSC showed enhanced osteogenesis, albeit with some interpatient variability. In addition, the mRNA expression of Osteopontin, an established marker of mature osteoblasts, was significantly upregulated in tMN BMSC compared to Healthy BMSC, whereas there was no difference in the expression of *RUNX2* (Fig. S14C–D).

### Senolytic therapies can reduce senescence burden and restore defective adipogenic and osteogenic differentiation of tMN BMSC

**Fig. 7:**
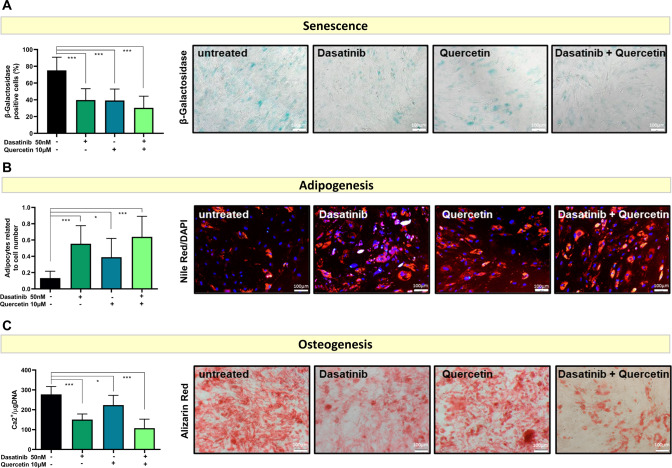
In vitro culture with senolytic agents restores tMN BMSC differentiation capacity. **A** Cellular senescence assessed by percentage of β-Galactosidase-stained cells at passage 3 of tMN BMSC (*n* = 3 per each condition). Representative micrographs of β-Galactosidase-stained cells; **B** tMN BMSC were cultured under adipogenic inductive conditions for 4 weeks either with or without senolytic drug and adipogenesis was assessed by quantification of Nile red-stained lipid droplets, relative to total number of DAPI stained cells (*n* = 3 per each condition). Representative micrographs of Nile red stained lipids; **C** tMN BMSC were cultured under osteogenic inductive conditions for 4 weeks either with or without senolytic drug and quantification of mineral content was assessed by measuring the concentration of Ca^2^ in an acid-solubilized matrix and normalizing them to the total DNA (*n* = 3 per each condition). Representative photos of mineral deposits stained with Alizarin Red. All bars indicate mean, and all error bars indicate SD. Scale bars indicate 100 µm. Mann–Whitney test was used to detect statistically significant differences between cohorts. Asterisks display *P* values * *P* < 0.05, *** *P* < 0.001.

We have shown that tMN BMSC are highly senescent yet metabolically highly active and exhibit defective adipogenesis and osteogenic differentiation capacity. Hence, we assessed if senescence drives aberrant differentiation potential of tMN BMSC. Senolytic agents Dasatinib (75 ± 16 vs. 40 ± 14%, *P* < 0.001), Quercetin (75 ± 16 vs. 39 ± 14%, *P* < 0.001) alone or in combination (75 ± 16 vs. 30 ± 14%, *P* < 0.001) significantly reduced senescence burden of tMN BMSC (Fig. 7A). Strikingly, senolytics therapy restore adipogenic differentiation as reflected by sixfold increase in adipogenic potential (0.13 ± 0.08 to 0.64 ± 0.25, *P* < 0.001) (Fig. 7B). Moreover, senolytics also reduces aberrantly excessive osteogenic potential to near normal (277 ± 40 vs. 108 ± 45 Ca^2+^/µg DNA, *P* < 0.001) (Fig. 7C). Together, these results demonstrate that senolytics therapies can restore aberrant differentiation potential of tMN BMSC.

### Long-term irreversible damage to BM microenvironment is induced by cytotoxic therapies

**Fig. 8:**
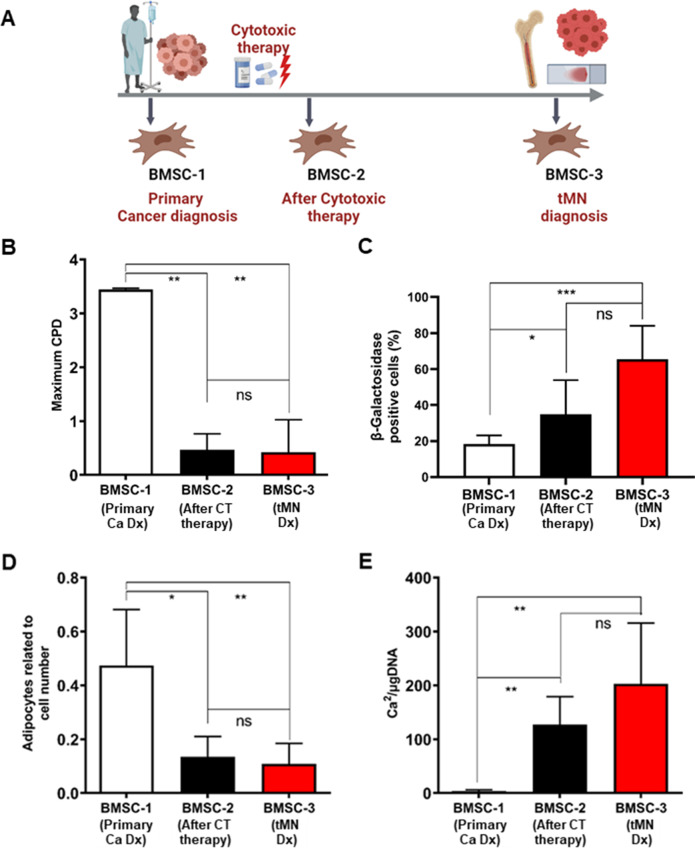
Irreversible long-term BMSC damage is induced by CT in tMN patients. **A** Schematic of tMN patient journey and serial BMSC collection such as at the time of primary cancer (BMSC-1), after completion of initial CT (BMSC-2) and at diagnosis of tMN (BMSC-3); **B** proliferation potential assessed by Maximum CPD; **C** quantification of senescence levels assessed by β-Galactosidase-stained cells; **D** adipogenic differentiation of BMSC quantified at 21 days by Nile red-stained lipid droplets; **E** quantification of mineral content by measuring the concentration of Ca^2^ in an acid-solubilized matrix and normalizing them to the total DNA. The results from three tMN patients (*n* = 3) at different time are shown; All bars indicate mean, and all error bars indicate SD. Scale bars indicate 100 µm. Mann–Whitney test was used to detect statistically significant differences between cohorts. Asterisks display *P* values *P < 0.05, **P < 0.01, ***P < 0.001.

In our study, tMN BMSC were significantly abnormal compared to pMN and pMN+Ca and the only difference between these groups was prior CT exposure. To test the hypothesis that prior exposure to CT leads to long-term damage to BM microenvironment, we assessed the proliferation, cellular senescence, and differentiation potential of serial BMSC of tMN patients (*n* = 3) isolated at the time of primary cancer, after completion of initial CT and at tMN diagnosis (Fig. 8A).

There was striking reduction in proliferative capacity of BMSC, as assessed by CPD, after 2–3 months of CT compared to sample collected at diagnosis of primary cancer (0.47 ± 0.29 vs. 3.45 ± 0.02; *P* = 0.009) (Fig. 8B and Fig. S15A). Moreover, the reduced proliferative potential of BMSC was observed at tMN diagnosis (0.47 ± 0.29 vs. 0.43 ± 0.60 CPD; *P* = 0.993) (Fig. 8B).

Number of senescent cells from BMSC following CT were significantly higher (35 ± 19%) than among the BMSC from primary cancer (18 ± 5%; *P* = 0.01) and remained high at tMN diagnosis (18 ± 5% vs. 65 ± 19%; *P* = 0.001) (Fig. 8C; Fig. S15B).

Moreover, adipogenic potential was significantly reduced following CT compared to primary cancer diagnosis (0.13 ± 0.08 vs. 0.47 ± 0.21; *P* = 0.010). Reduced adipogenesis persisted at tMN diagnosis (0.13 ± 0.08 vs. 0.11 ± 0.08; *P* = 0.463) (Fig. 8D and Fig. S15C). In contrast, osteogenic potential was significantly increased following CT compared to primary cancer diagnosis (127.1 ± 51.85 vs. 3.54 ± 2.52 Ca^2+^/µg DNA; *P* = 0.002) and persisted at tMN diagnosis (127.1 ± 51.85 vs. 202.7 ± 112.9 Ca^2+^/µg DNA; *P* = 0.244) (Fig. 8E and Fig. S15D). Together these data suggest that CT led to irreversible damage to BMSC, which was evident many years prior to tMN diagnosis.

## Discussion

Currently genetic and epigenetic alterations in HSC are considered to drive myeloid malignancies [2, 3]. Although, the microenvironment regulates the fate and maturation of normal hematopoiesis [32], its role in the pathogenesis of myeloid cancer remains an area of active research. To our knowledge, this is the first comprehensive ex-vivo study examining the role of the microenvironment in tMN patients with clinical curation and age-matched controls. Significantly, we demonstrate several novel findings: (i) tMN stromal cells are not only distinct from healthy age-matched stroma but are also distinct from other primary myeloid neoplasms, developing apart from cytotoxic exposure. (ii) tMN stromal cells are highly senescent with a characteristic flattened morphology, defective regenerative capacity, high p21 and β-Galactosidase expression and evidence of an active and ongoing senescence-associated secretory response. (iii) They exhibit a selective defect in adipocyte differentiation that was experimentally phenocopied by treating healthy BMSC with senescence-secreted cytokines IL-1β and IFNγ. (iv) tMN stroma is dormant, non-apoptotic but highly metabolically active with a bioenergetic shift toward glycolysis resembling Warburg metabolism. (v) Serial sampling shows that exposure to DNA damaging agents leads to pro-inflammatory stromal defects evident many years before the onset and diagnosis of tMN, an unexpected finding with repercussions for patients treated with chemotherapy or radiotherapy. (vi) Finally, we show that senolytic agents effectively reduced the senescence burden and restored differentiation potential, indicating a possible role of senolytic therapies in modulating tMN long term.

Cellular fate following genotoxic stress is governed by several factors including the competency of the DDR mechanism and intact *TP53* function. Based on our signalling results, tMN is associated with delayed recruitment of upstream kinases such as ATM and CHK2. Classically, fibroblasts unable to repair DNA damage undergo apoptosis or cellular senescence. Here, tMN BMSC exhibited multiple features of senescence rather than apoptosis and possibly are linked to a delayed and sub-optimal DNA damage response hampering efficient repair. Tumor cells harboring wildtype *TP53* are more likely to senesce in response to genotoxic therapies [33, 34], whereas cells carrying mutant *TP53* (Li-Fraumeni syndrome) overcome senescence readily and are prone to develop cancer [35]. Following genotoxic stress, active *TP53* halts cell-cycle progression via key mediator of senescence, cyclin-dependent kinase inhibitor *CDKN1A* (p21). *CDKN1A* expression was significantly higher in tMN as compared to Healthy BMSC and increased up to 30-fold in tMN after irradiation. Together, these data suggest that delayed and incomplete DNA repair leads to high and sustained level of p21 driving cellular commitment toward senescence and thus provide evidence that the p53-p21 pathway is still intact in tMN BMSC.

We note a strong overlap between pro-inflammatory cytokines secreted by tMN stroma cells and published literature for a senescence-secretory phenotype [27]. The extent to which senescence-related cytokines contribute to the pathogenesis of tMN is an intriguing possibility and should be explore in future studies, especially in light of the data in Fig. 7 which shows senescence is acquired early in the patient journey, soon after cytotoxic treatment. Our data suggest the secretome is modifying stromal fate, in that senescence-associated cytokines IL-1β, IFNγ, IL-6, IL-7 and IL-15 significantly overlap with known inhibitors of adipogenesis [36], were abundantly secreted and strongly inhibited adipogenesis (IL-1β, IFNγ), in agreement with previous studies [37–43].

Despite their dormancy, we were surprised by the hyper-energetic glycolytic state observed in tMN stroma. In a transgenic Eµ-myc lymphoma model, senescence-inducing chemotherapy led to a hyper-catabolic phenotype with increased glycolysis and ATP production [44]. Similarly, irradiation-induced senescence has been linked to reduced tricarboxylic acid activity and a shift toward glycolysis [45]. Future work should examine flux pathways in senescent vs. non-senescent BMSC [44].

Senolytics, including Dasatinib and Quercetin, have been shown to selectively eliminate senescent cells from both human and mouse [46–50] with evidence that sufficient restoration of function may occur without eliminating all senescent cells [46, 48, 51]. Indeed, in our study, senolytics restored the defect in adipogenesis and osteogenic differentiation in tMN. These results flag further research into senolytics for regenerative approaches.

Strikingly, the reduced adipogenic differentiation of BMSC was evident within few months following cytotoxic therapies in patients subsequently progressing to tMN, as was the upregulation of β-Galactosidase. This study illustrates the abrupt and dramatic effect of in vivo cytotoxic exposure on the differentiation capacity of primary patient BMSC. It is possible but not prospectively proven, that the BMSC-derived highly pro-inflammatory SASP could initiate or promote a form of clonal hematopoiesis, eventually progressing to tMN. One limitation of our study is that the serial BMSC studies (Fig. 8), while convincingly show evidence of time-dependent causality before and after exposure to CT, were obtained exclusively from patients with multiple myeloma. Future studies should explore other primary cancer diagnoses (such as prostate or breast cancer) and determine whether specific types of cancer and cytotoxicity result in specific BMSC phenotypes.

In summary, our study provides comprehensive phenotypic, functional, and molecular profiling of tMN BMSC compared to carefully curated samples from pMN, pMN+Ca (without prior exposure to CT) and age-matched healthy control. Together, our data signals a precision approach to tMN vs. other blood cancers and underscores the presence of a pro-inflammatory but eminently targetable stromal milieu that may underly tMN pathogenesis.

## Supplementary information

Supplementary material

## Data Availability

All data generated or analyzed during this study are included in this published paper and its Supplementary Information.
