# Supplementary material for: A senescence stress secretome is a hallmark of therapy-related myeloid neoplasm stromal tissue occurring soon after cytotoxic exposure
Source: Leukemia. 2022 Aug 29;36(11):2678–89. doi: 10.1038/s41375-022-01686-y (PMC9613466; doi:10.1038/s41375-022-01686-y)
Supplement: Supplementary file 1 — Supplementary material [file 41375_2022_1686_MOESM1_ESM.docx]

**Supplementary Information**

**This section includes:**

1. **Supplementary Methods including references**
2. **Tables S1- S3**
3. **Supplementary Figures S1-S15**

**Supplementary Methods**

***Patient samples:*** tMN is a rare disease, hence samples are very limited. Number of individual patient sample used are provided with each experiment results.

***Isolation, expansion, culture conditions and characterization of BMSC:*** BMSC were isolated and expanded from BM-mononuclear cells (MNC). In brief, primary cultures were established by culturing 3x10^4^ MNC per cm^2^ in alpha Modification of Eagle’s Medium (αMEM) supplemented with 20% fetal calf serum (FCS), 2mM α-glutamine, 1mM sodium pyruvate, 100μM α-ascorbate-2-phosphate, and penicillin (50units/mL)/ streptomycin (50µg/mL) (Sigma-Aldrich, St Louis, MO, USA) at 37ºC humidified atmosphere in the presence of 5% CO_2._ Non-adherent hematopoietic cells were removed, and medium was replaced twice per week. Upon achieving 80%–90% confluency, cells were detached using TrypLE Express (Life Technologies, Carlsbad, CA, USA), counted, reseeded at a concentration of 1x10^4^ cells per cm^2^, and further expanded in αMEM growth medium supplemented with 10% FCS. Assays were performed on BMSC between the second and fourth passage. To meet the criteria of the International Society for Cellular Therapy^1^ and to exclude hematopoietic contamination, BMSC at passage 3 were analyzed by flow cytometry for the expression of CD73, CD90, CD105, B478 and CD146 (Cat# 550256, 555594, 555690, 561433, 550314; BD Biosciences), and negativity to CD34 and CD45 expression (Cat# 348057; BD Biosciences), (**Figure S2A**). Colony-forming unit fibroblasts (CFU-F) were assessed by culturing MNC at densities of 2x10^4^-6x10^4^ cells per well of 24-well plate in triplicate, over 14 days as previously described^2^.

***Cytoskeleton assay:*** BMSC cytoskeleton was assessed using tetramethyl rhodamine (TRITC)- labelled Phalloidin (Cat# P1951; Sigma-Aldrich, St Louis, MO, USA) and counterstaining the nuclei with 4′,6-diamidino-2-phenylindole (DAPI). BMSC were washed with PBS and fixed in 4% paraformaldehyde for 30 minutes, then wash three times with PBS + 0.1% Tween 20, followed by 2 hours incubation with TRITC- labelled Phalloidin, 1:2000 at room temperature. The nuclei were counterstained with DAPI and viewed under fluorescent microscopy. Image analysis and quantification was performed using ImageJ Software (National Institutes of Health and the Laboratory for Optical and Computational Instrumentation (LOCI, University of Wisconsin, USA).

***Proliferative potential of BMSC:*** BMSC cultures were initiated at passage 0 and cells were reseeded at fixed density (8x10^3^cells per cm^2^) until population expansion had ceased. The formula logN/log2 was used to calculate the cell population doublings (PD) at each passage. N was the ratio of cells counted at the end of a particular passage and the number of cells initially plated. Days for one PD at a given passage was based on the length of culture in days divided by the number of the respective cell PD. Cumulative PD (CPD) were regarded as total cell expansion. ***Long term culture-initiating cells (LTC-IC) assay:*** To assess hematopoietic supporting capacity of BMSC, LTC-IC assays were performed. Briefly, feeder layer cells (7.5x10^4^ stromal cells per well of 24-well plate) were cultured until at least 80% confluency was reached and then irradiated (30Gy). Subsequently, purified CD34^+^ HSC (1x10^4^ CD34^+^ per well) were re-suspended in 500μl LTC medium H5100 (StemCell Technologies, BC, Canada) with freshly added Hydrocortisone (StemCell Technologies, BX, Canada; final concentration 10^-6^ M) and plated on pre-established stromal layers. Cultures were maintained for 5 weeks at 37ºC, 5% CO_2_ with weekly changes of half medium. Afterwards, cells from each well were harvested and were plated in methylcellulose medium H4230 (StemCell Technologies, BC, Canada) with 5-growth factor mix (G-CSF, GM-CSF, SCF, IL-3, IL-6; Miltenyi Biotech, Bergisch Gladbach, Germany) resuspended in serum deprived media and cultured for 14 days at 37ºC, 5% CO_2_. After two weeks, colonies were scored based on their morphology under a light microscope.

***Apoptosis assay:*** The number of apoptotic cells at passage 3 was quantified using the Annexin-V-PE Kit (Cat# 640908; BD Biosciences, San Jose, CA USA) according to the manufacturer’s instructions. Early apoptotic cells were defined as Annexin-V-positive, 7-Aminoactinomycin D (7AAD) negative cells. The analyses were performed on an FACS Canto II flow cytometer (BD Biosciences, San Jose, CA, USA).

***Analysis of cellular senescence:*** Cellular senescence of BMSC at passage 3 was evaluated by assessing expression of β-Galactosidase (Cell Signaling Technology, Danvers, MA, USA) according to the manufacturer’s specifications. Number of β-galactosidase positive cells (blue) was evaluated under phase-contrast microscope and normalized to DAPI stained nuclei per four fields of view in triplicate wells.

***Transcriptome sequencing:*** Whole transcriptome analysis was performed on BMSC isolated from tMN (n = 3) and compared with age-matched healthy controls (n = 3), pMN (n = 3), and pMN+Ca (n = 3). Transcriptome sequencing libraries were prepared with Illuimina TruSeq Total RNA protocol (Illumina, San Diego, CA, USA) for 150bp paired-end sequencing on a NextSeq500 instrument.

Adapters were trimmed off raw sequencing read pairs using cutadapt (v2.10) and aligned to version b37 of the human genome with STAR (v2.7.2c), using in-house scripts that made use of 10bp UMIs in order to remove PCR duplicate fragments (umitools v1.0.0, sambamba v0.8.0, bedtools v2.27.1). Counts of fragments under gene models were carried out through STAR quantmode GeneCounts, mapping statistics were quantified using Picard (v2.16.0) and samtools (v1.9). Gene expression was analyzed via the BioConductor R (v4.0.3) package edgeR (3.30.3). Genes were filtered out as 'unexpressed' if expression was not greater than 1 count per million for all samples in at least one of the four groups of samples. Finally, gene expression was normalized using the trimmed mean of medians method. Only genes with adjusted *P*-value (based on false discovery rate, FDR) less than 0.1^3^, nominal *P* < 0.05, were considered as statistically significant. Heatmaps were generated using the pheatmap R package.

***Identification of senescence gene expression based on β-Galactosidase levels:*** Gene expression was analyzed similar to the Transcriptome sequencing section above. Briefly, the differential gene expression was analyzed based on the β-Galactosidase value via the BioConductor R (v4.0.3) package edgeR (3.30.3). Only genes with FDR *P* < 0.05 derived from edgeR analysis were considered as statistically significant. The Pearson product-moment correlation coefficient was calculated for each gene against the β-Galactosidase to measure the strength of a linear association.

***Gene set enrichment analysis:*** Gene-set enrichment analysis (GSEA) was performed using Broad Institute GSEA software version 4.0 and Molecular Signature Database (MSigDB) version 7.2. MSigDB is a collection of annotated gene sets for use with the GSEA software. Briefly, the pre-ranked gene list ranked by edgeR was used to perform GSEA. The pre-ranked gene list was used to perform gene-set enrichment analysis against MSigDB hallmark gene sets (H), curated gene sets (C2), and gene ontology (GO) gene sets (C5). The default setting was used when performing the GSEA analysis. Only gene sets with FDR *P* < 0.1 were considered as statistically significant enriched in the gene list.

***Quantitative real-time PCR (qRT-PCR):*** Genes differentially expressed in the transcriptome analysis were validated in the expanded cohort of samples using quantitative real-time PCR. Total RNA isolated from BMSC was extracted using the RNeasy Plus Micro kit (Qiagen, Hilden, Germany) and reverse transcribed into cDNA using SuperScript IV VILO (Thermo Fisher, Waltham, United States) according to the manufacturer’s instructions. qRT-PCR target transcripts were amplified with the primers listed in **Table S3**, using SYBR Green (Bio-Rad, Hercules, United States). All qRT-PCR reactions were performed in triplicate with changes in gene expression made relative to the housekeeping gene *GAPDH* or β-actin as specified in the figures, using the 2-ΔCT method.

***Cytokines/chemokines (C/CH) levels:*** Levels of 38 C/CH, including 14 C/CH associated with senescence-associated secretory phenotype (SASP) and 12 C/CH associated with adipogenesis from BMSC-conditioned media were assessed using Merck HCYTOMAG-60K MILLIPLEX MAP Human Cytokines/Chemokines magnetic bead panel (Merck, St Louis, Missouri, USA) and the MagPix 4.2 instrument (Luminex, Austin, TX, USA) using Luminex xMAP technology according to the manufacturer’s protocol. Assay sensitivity ranged from 0.4 to 26.3pg/ml, depending on the analyte. BMSC- conditioned media was collected after culture of confluent BMSC in serum free α-MEM media for 24 hours.

***Alkaline comet assay:*** Comet assay was performed under alkaline electrophoresis conditions at 20V/300mA for 30 minutes following the manufacturer’s instructions (Abcam, Cambridge, United Kingdom) at baseline and after 24 hours of 0.1µM and 1µM Doxorubicin (Sigma Aldrich, Australia) treatment.

***Reactive Oxygen Species (ROS) level:*** ROS production was determined by measuring the formation of fluorescent dichloro fluorescein (DCF) at an Excitation 485 and Emission 535 nm using Cellular ROS Assay Kit (Abcam, Cambridge, United Kingdom). The final value was obtained by calculating the ratio of fluorescence intensity at specific time point and DAPI stained nuclei.

***DNA damage repair potential following irradiation:*** DNA damage repair potential of BMSC following sublethal dose of radiotherapy were assessed by phosphorylation of γH2AX, a dsDNA break-associated histone. BMSC were seeded in 24-well plates (1x10^4^ per well) and cultured under normal conditions until 80—90% confluency was reached before exposing them to 20Gy irradiation using an X-ray irradiator (RS 2000 X-ray irradiator, Rad Source Technologies, Suwanee, GA, USA). DNA damage response was assessed in non-irradiated and at 0, 1, 3, 5 and 8 hours post irradiation. Briefly, cells were washed in PBS (phosphate-buffered saline; Sigma-Aldrich, St Louis, MO, USA) and permeabilized in 0.25% triton X-100 (Sigma-Aldrich, St Louis, MO, USA) in PBS for 30 minutes, followed by two PBS washes and incubation in blocking buffer (1% BSA, 5% normal goat serum, 5% normal rabbit serum in PBS) for 1 hour. Cells were then stained against γH2AX using the anti-gamma H2AX (phospho S139) antibody (Cat# 05-636; Merck, St Louis, Missouri, USA) for 2 hours, according to the manufacturer’s protocol. Anti-mouse Alexa 488 (Cat# LTSA- 11017; Thermo Fisher, Waltham, MA, USA) was used as the secondary antibody for which incubation time was 1 hour. Number of γH2AX foci was evaluated under phase-contrast microscope and normalized to DAPI stained nuclei per four fields of view in triplicate wells once cells were stained against γH2AX.

***DNA damage induction by in vitro exposure to Doxorubicin:*** Healthy and tMN BMSC were seeded in 24-well plates (1x10^4^ per well) and cultured under normal conditions until 80—90% confluency was reached before *in vitro* administration of 0.1µM and 1µM of Doxorubicin for 4, 12 and 24 hours. Following drug exposure, cells were washed and stained with γH2AX.

***DNA damage repair potential following in vitro exposure to Doxorubicin:*** Healthy and tMN BMSC were seeded in 24-well plates (1x10^4^ per well) and cultured under normal conditions until 80—90% confluency was reached before *in vitro* administration of 0.1µM Doxorubicin for 24 hours. Following drug exposure, cells were washed and cultured further in drug free media for 1, 3, 5, 8, 12 and 24 hours. Following each time cells were stained with γH2AX.

***Western immunoblotting:*** Response to *in vitro* DNA damaging radiotherapy was assessed in tMN and age matched Healthy BMSC by assessing phosphorylation of early responders such as ataxia telangiectasia mutated (ATM) and chekpoint-2 (CHK2), and effector proteins (p53 and p21) of DNA damage repair. Phosphorylated and total ATM, CHK2, p53 and p21 were assessed at 0.5, 1, 3, 5, 8 and 24 hours following 20 Gy irradiation in Healthy and tMN BMSC and compared with baseline (non-irradiated cells). Beta-actin was used as the protein loading control. The following primary antibodies were used: Anti–phospho-ATM (Cat# AF1655, R&D Systems, Minneapolis, MN, USA); anti-phospho-p53 (Cat# 9284, Cell Signalling Technology, Beverly, MA, USA); anti-phospho-CHK2 (T68) (Cat# 2661, Cell Signalling Technology); anti-ATM (Cat# 1873, Cell Signalling Technology); anti–p53 (Cat# sc-126, Santa Cruz Biotechnology, Dallas, Texas, USA); anti-p21 (Cat# 5567430, BD Bioscience, Franklin Lakes, NJ, USA); anti-β-actin (Cat# A1978, Sigma Aldrich, St. Louis, MO, USA). Thirty micrograms of protein samples were denatured and electrophoresed on Mini or Midi TGX- Stain-free precast gels (Bio-Rad, Hercules, CA, USA) at 110V for 60-80 minutes in SDS-PAGE running buffer (250mM Tris, 192 mM Glycine, 0.06% SDS). Proteins were then transferred onto PVDF membrane (Bio-Rad) using a Trans-Blot Turbo (Bio-Rad) according to the manufacturer’s instructions. Membranes were then incubated in blocking buffer [TBS-T (20 mM Tris, 150mM NaCl pH 7.4, 0.05% Tween- 20)] containing 5% BSA (Sigma Aldrich) for 1 hour at room temperature, followed by incubation with primary antibody for 1 hour at room temperature or overnight at 4°C. Membranes were washed in TBST for 4 x 15 minutes, followed by incubation with horseradish peroxidase (HRP), alkaline phosphatase (AP) or Cy5-conjugated secondary antibodies for 1 hour at room temperature. Following membrane washing in TBST for 4 x 10 minutes, Enhanced Chemi-Luminescence (ECL) reagent (Pierce Chemical Co., Rockford, IL, USA) or Enhanced Chemifluorescence (ECF) reagent (Cytiva, Marlborough, MA, USA) were used for immunoblot development. Fuji LAS4000 System (GE Healthcare) or Typhoon FLA 9000 (Cytiva) were used for detecting luminescence or fluorescence signals.

***Real-Time ATP Rate Assay******:*** tMN and Healthy BMSC were seeded into the Seahorse XF96 Cell Culture Microplates (Agilent Technologies, Santa Clara, CA, USA) at the cell density of 10^4^ cells/well and cultured until confluence. Prior to the start of the assay, a sensor cartridge was hydrated in Seahorse XF Calibrant following the manufacturer’s instruction. Immediately before the experiment, growth media was replaced with Seahorse XF DMEM Medium, containing 10mM glucose, 2mM L-glutamine and 1mM sodium pyruvate. After calibration of the Seahorse XF96 Analyzer, the sensor cartridge was removed from the instrument and Seahorse XF96 Cell Culture Microplate was inserted. A baseline and over time measurements of the oxygen consumption rate (OCR) and the extracellular acidification rate (ECAR) was taken, and then an inhibitory analysis was performed using injections of mitochondrial inhibitors Oligomycin at 1μM, and Rotenone+ Antimycin A at 1μM. For the normalization of the Seahorse data, we used a PicoGreen dsNDNA quantification kit (Thermo Fisher, Waltham, MA, USA). Results were exported by using the Seahorse Wave 2.4 XF-96 software and the data obtained were expressed relative to the total DNA per well and expressed in pmol/min/μg DNA.

The effect of glycolysis inhibitor on ATP production was also evaluated by culturing tMN and Healthy BMSC with 1mM or 2mM of 2-Deoxy-D-glucose (2DG; Sigma Aldrich, Australia) for 72 hours. Different concentrations of this drug were tested first to assess its effect on BMSC viability without toxicity to cells.

For *in vitro* adipogenic assays, OXPHOS inhibitor (IACS-010759; Selleckchem, Australia) at a concentration of 100nM, cytokines (IL-1β, IL-6, IL-7, IL-13, IL-15, IFNα and IFNγ; Miltenyi Biotec, Australia) at a concentration of 20ng/ml and 2DG (Sigma Aldrich, Australia) at concentration of 1mM or 2mM were added into adipogenic induction media twice a week for 14 days.

To assess the *in vitro* effect of senolytic therapy on tMN BMSC differentiation capacity, Dasatinib (Bristol Myers Squibb, USA) at concentration of 50nM and Quercetin (Sigma Aldrich, Australia) at concentration of 10µM were added two times a week for 21 days to adipogenic and osteogenic induction media.

***BMSC differentiation:*** *In vitro* expanded BMSC at passage three were further cultured in osteoblast and adipocyte differentiation media for up to 28 days as previously described^2^. Differentiation potential was assessed by the cytochemical staining (Alizarin Red for mineralized bone matrix formation and Oil Red for lipid formation) (Sigma-Aldrich, St Louis, MO, USA) and quantification of positive cells (mineral quantification by Calcium Arsenazo III (Pointe Scientific, Lincoln Park, Michigan, USA) normalized to DNA content with a PicoGreen dsDNA quantification kit (Thermo Fisher, Waltham, MA, USA)) and lipid quantification by Nile Red normalized to DAPI (Sigma-Aldrich, St Louis, MO, USA).

**Supplementary References**

1. Horwitz, E.M., et al., *Clarification of the nomenclature for MSC: The International Society for Cellular Therapy position statement.* Cytotherapy, 2005. **7**(5): p. 393-5.

2. Gronthos, S., et al., *Molecular and cellular characterisation of highly purified stromal stem cells derived from human bone marrow.* Journal of Cell Science, 2003. **116**(9): p. 1827-1835.

3. Anders, S. and W. Huber, *Differential expression analysis for sequence count data.* Genome Biology, 2010. **11**(10): p. R106.

**Supplementary Tables**

**Table S1. Demographics and clinical characteristics of pMN, pMN+Ca and tMN patients**

| Variable | pMDS/pAML (pMN)  (n = 7) | pMN+Ca (n = 10) | tMN (n = 18) |
| --- | --- | --- | --- |
| Median age at first malignancy | NA | 68 (48-82) | 66 (46-81) |
| Median age at myeloid malignancy | 70 (64-86) | 75 (64-87) | 74 (58-85) |
| Median latency from first  malignancy to myeloid | NA | 3 (0-19) | 5 (1-19) |
| Median latency from last dose of first malignancy treatment to myeloid malignancy | NA | NA | 3 (0.3-18) |
| Gender (Male/Female) | 7/3 | 7/3 | 9/9 |
| Myeloid malignancy diagnosis: |  |  |  |
| MDS-MLD/MDS-RS-MLD | 1 (14%) | 4 (40%) | 8 (44%) |
| MDS-EB-1/EB-2 | 3 (43%) | 3 (30%) | 5 (28%) |
| MDS/MPN overlap | 2 (29%) | 2 (20%) | 2 (11%) |
| AML | 1 (14%) | 1 (10%) | 3 (17%) |
| Primary malignancy diagnosis: |  |  |  |
| Non-Hodgkin Lymphoma | NA | 1 (10%) | 4 (22%) |
| Multiple Myeloma | NA | 1 (10%) | 4 (22%) |
| Breast Cancer | NA | 0 | 3 (17%) |
| Prostate Cancer | NA | 2 (20%) | 2 (11%) |
| Rectum Cancer | NA | 1 (10%) | 1 (6%) |
| Autoimmune Disease- Psoriasis | NA | 0 | 2 (11%) |
| Other malignancies | NA | 5 (50%) | 2 (11%) |
| Treatment type of first malignancy: |  |  |  |
| CT only | NA | NA | 7 (39%) |
| RT only | NA | NA | 4 (22%) |
| CT & RT | NA | NA | 7 (39%) |
| Karyotype: |  |  |  |
| Normal | 6 (86%) | 5 (50%) | 7 (39%) |
| Complex (≥3 abnormalities) | 1 (14%) | 1 (10%) | 3 (17%) |
| Other abnormalities | 0 | 3 (30%) | 8 (44%) |
| Not performed | 0 | 1 (10%) | 0 |

Abbreviations: pMDS, primary myelodysplastic syndromes; pAML, primary acute myeloid leukemia; pMN, primary myeloid neoplasms; pMN+Ca, primary myeloid neoplasms + Cancer; tMN, therapy related myeloid neoplasms; 1°, primary; NA, no applicable; MDS-MLD, MDS with multilineage dysplasia; MDS-RS-MLD, MDS with ring sideroblasts with MLD; MDS-EB-1, MDS with excess blasts-1; MDS-EB-2, MDS with excess blasts-2; myelodysplastic/ myeloproliferative neoplasms overlap syndrome (MDS/MPN overlap) include chronic myelomonocytic leukemia and atypical chronic leukemia. Other malignancies include sarcoma, melanoma, cancers of lung/ovary/ neochroid/ bowel/colon/ throat); CT, chemotherapy; RT, radiotherapy.

**Table S2. Treatment details for primary diseases in tMN patients**

| ID | Primary disease | Previous therapy |
| --- | --- | --- |
| tMN_1 | NHL | Combination chemotherapy including cyclophosphamide, vincristine, prednisolone, and local radiotherapy |
| tMN_2 | MM | Melphalan and prednisolone and local radiotherapy |
| tMN_3 | Sarcoma | Ifosfamide and Doxorubicin, local radiotherapy, resection surgery |
| tMN_4 | MM | Vincristine, doxorubicin and dexamethasone, local radiotherapy, autologous stem cell transplant and lenalidomide maintenance |
| tMN_5 | Rectum Ca and SLE | Immunomodulatory therapies including azathioprine, mycophenolate, cyclosporine for SLE. Chemoradiotherapy and surgical resection for cancer |
| tMN_6 | MM | Combination chemotherapy of dexamethasone, thalidomide, cisplatin, doxorubicin, cyclophosphamide and etoposide followed by autologous stem cell transplant and thalidomide maintenance |
| tMN_7 | Breast Ca | Combination chemotherapy including adriamycin, cyclophosphamide, methotrexate, fluorouracil, local radiotherapy and left mastectomy followed by hormonal therapy tamoxifen |
| tMN_8 | Prostate Ca | Local radiotherapy |
| tMN_9 | Psoriasis | Weekly methotrexate for 6-7 years |
| tMN_10 | NHL | Combination chemotherapy including cyclophosphamide, vincristine, doxorubicin, prednisolone (R-CHOP). Repeated cycles of rituximab, dexamethasone, cytarabine, cisplatin (R-DHAP) followed by autologous stem cell transplant |
| tMN_11 | Throat Ca | Local radiotherapy |
| tMN_12 | NHL | Combination chemotherapy including cyclophosphamide, vincristine, doxorubicin, prednisolone (R-CHOP) and high dose methotrexate. Gemcitabine based chemotherapy for relapse |
| tMN_13 | Psoriasis | Weekly methotrexate for four years |
| tMN_14 | NHL | Combination chemotherapy including cyclophosphamide, vincristine, doxorubicin, prednisolone (R-CHOP) and intrathecal methotrexate |
| tMN_15 | MM | Combination chemotherapy including cyclophosphamide, vincristine, prednisolone and local radiotherapy |
| tMN_16 | Breast Ca | Surgical resection and local radiotherapy |
| tMN_17 | Prostate Ca | Surgical resection and local radiotherapy |
| tMN_18 | Breast Ca | Combination chemotherapy, local radiotherapy and left breast mastectomy |

Abbreviations: tMN, therapy related myeloid neoplasms; NHL, non-Hodgkins’s lymphoma; MM. multiple myeloma; Ca, cancer; SLE, systemic lupus erythematosus.

**Table S3. Primer sequences used for qRT-PCR**

| Gene symbol | Gene name | PrimerBank  ID | Amplicon  size | Sequence (5'-> 3') |
| --- | --- | --- | --- | --- |
| *FOS*-F | Fos Proto- Oncogene, AP-  1 Transcription Factor Subunit | 254750707c2 | 126 | GGGGCAAGGTGGAACAGTTAT |
| *FOS*-R |  |  |  | CCGCTTGGAGTGTATCAGTCA |
| *STC2*-F | Stanniocalcin 2 | 61676085c2 | 176 | ACAGGTTCGGCTGCATAAGC |
| *STC2*-R |  |  |  | GAGGTCCACGTAGGGTTCG |
| *IFITM1*-F | Interferon Induced Transmembrane  Protein 1 | 4504581a1 | 91 | CCAAGGTCCACCGTGATTAAC |
| *IFITM1*-R |  |  |  | ACCAGTTCAAGAAGAGGGTGTT |
| *CXCL12*-F | C-X-C Motif  Chemokine Ligand 12 | 296011022c1 | 88 | ATTCTCAACACTCCAAACTGTGC |
| *CXCL12*-R |  |  |  | ACTTTAGCTTCGGGTCAATGC |
| *CDKN1A*-F | Cyclin Dependent Kinase  Inhibitor 1A | 310832423c1 | 139 | TGTCCGTCAGAACCCATGC |
| *CDKN1A*-R |  |  |  | AAAGTCGAAGTTCCATCGCTC |
| *FGF2*-F | Fibroblast  Growth Factor 2 | 153285460c2 | 170 | AGTGTGTGCTAACCGTTACCT |
| *FGF2*-R |  |  |  | ACTGCCCAGTTCGTTTCAGTG |
| *UHRF2*-F | Ubiquitin Like With PHD And Ring Finger  Domains 2 | 324120894c1 | 184 | ATTGAGGACGTGTCTCGCAAA |
| *UHRF2*-R |  |  |  | GGTCTGGGCGAACTAGCAG |

**Supplementary Figures**

**
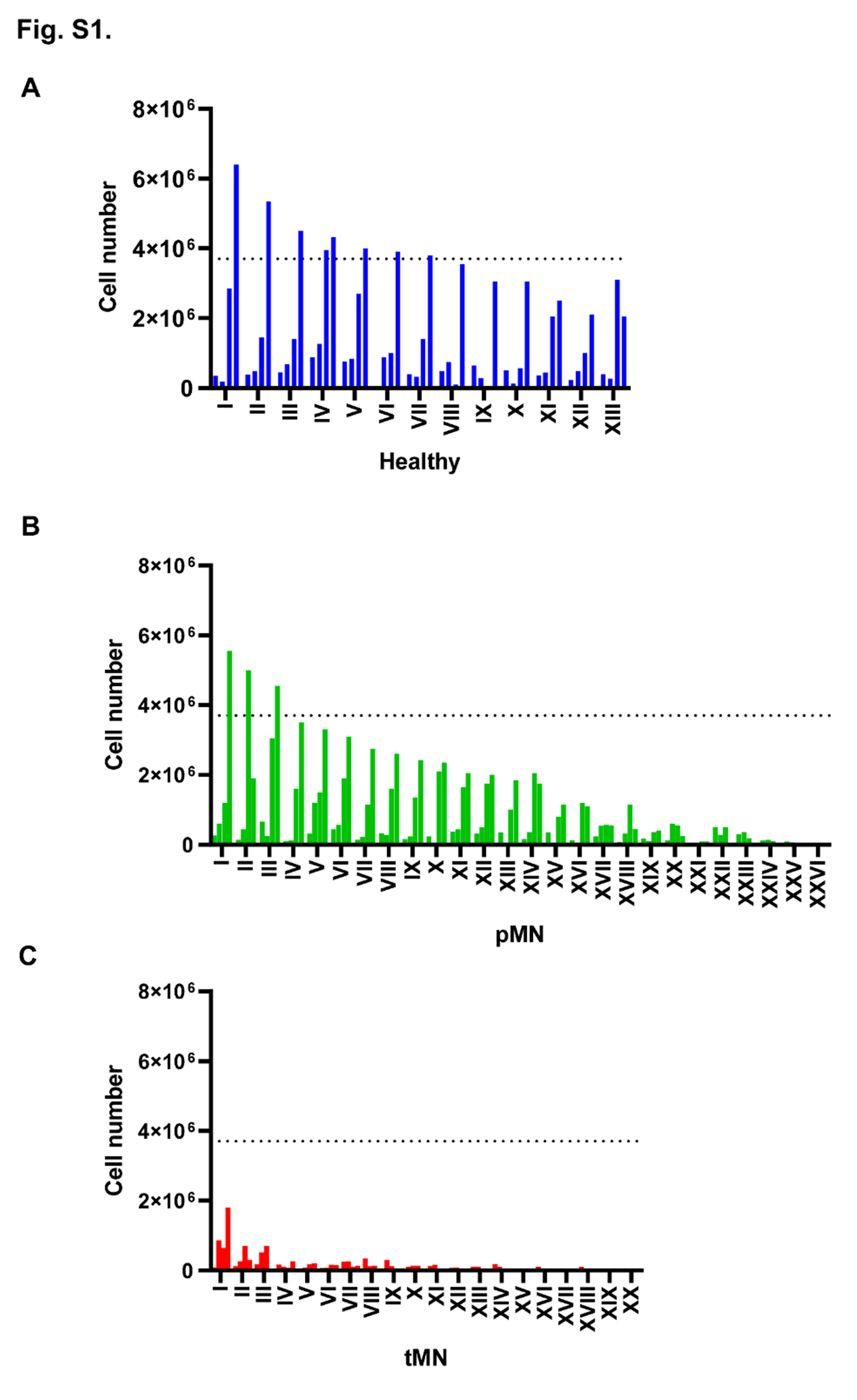
**

**Figure S1. Defective proliferative capacity of tMN compared to other myeloid neoplasm and Healthy BMSC in a pilot study cohort.** (A) Proliferation potential of Healthy (n = 13); (B) proliferation potential of pMN (n = 26); (C) proliferation potential of tMN (n = 20). Each bar represents number of cells at the end of each passage. Samples were expanded up to passage 4 (samples without bars did not grow).

**
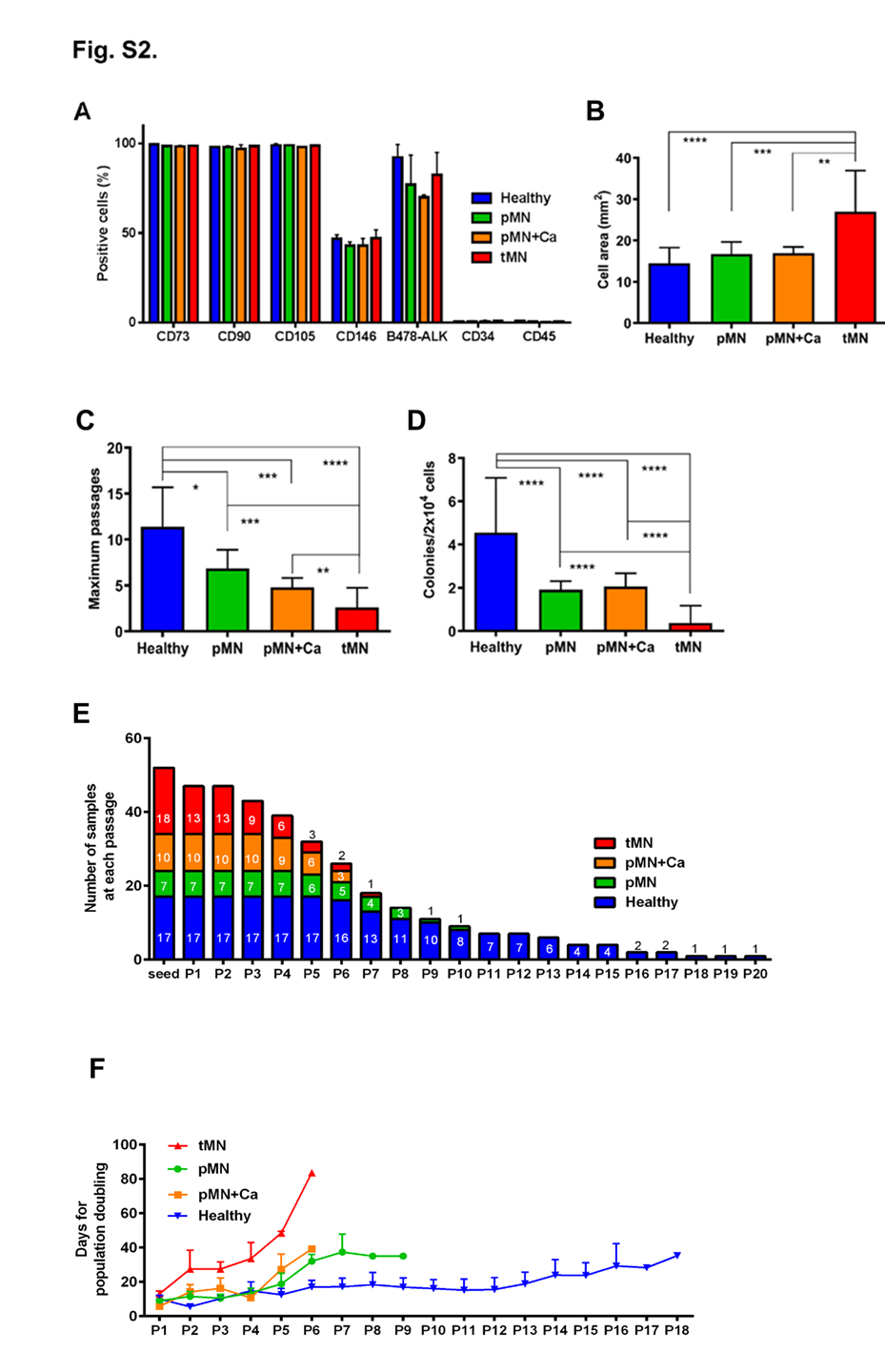
**

**Figure S2. tMN BMSC share common immunophenotypic features but exhibit defective colony forming capacity and slower growth kinetics compared to other MN and Healthy BMSC.** (A) Expression of positive and negative CD markers of culture-expanded at passage 3 (P3) BMSC analyzed by flow cytometry; (B) BMSC morphology assessed by the quantification of cells size normalised to DAPI-stained nuclei; (C) maximum number of passages; (D) colony forming unit-fibroblast (CFU-F) normalized to 2x10^4^ plated BM mononuclear cells; (E) stack graph illustrating number of samples with successful cultures at each passage; (F) days for population doublings (PD) through passages, based on the number of cells at the end of each passage compared with cells initially plated. Results shown in this figure are based on data from Healthy (n = 17), pMN (n = 7), pMN+Ca (n = 10) and tMN (n = 18). All bars indicate mean, and all error bars indicate SD. Mann-Whitney test was used to detect statistically significant differences between cohorts. Asterisks display *P*-values **P* < 0.05, ***P* < 0.01, ****P* < 0.001, **** *P* < 0.0001.

**
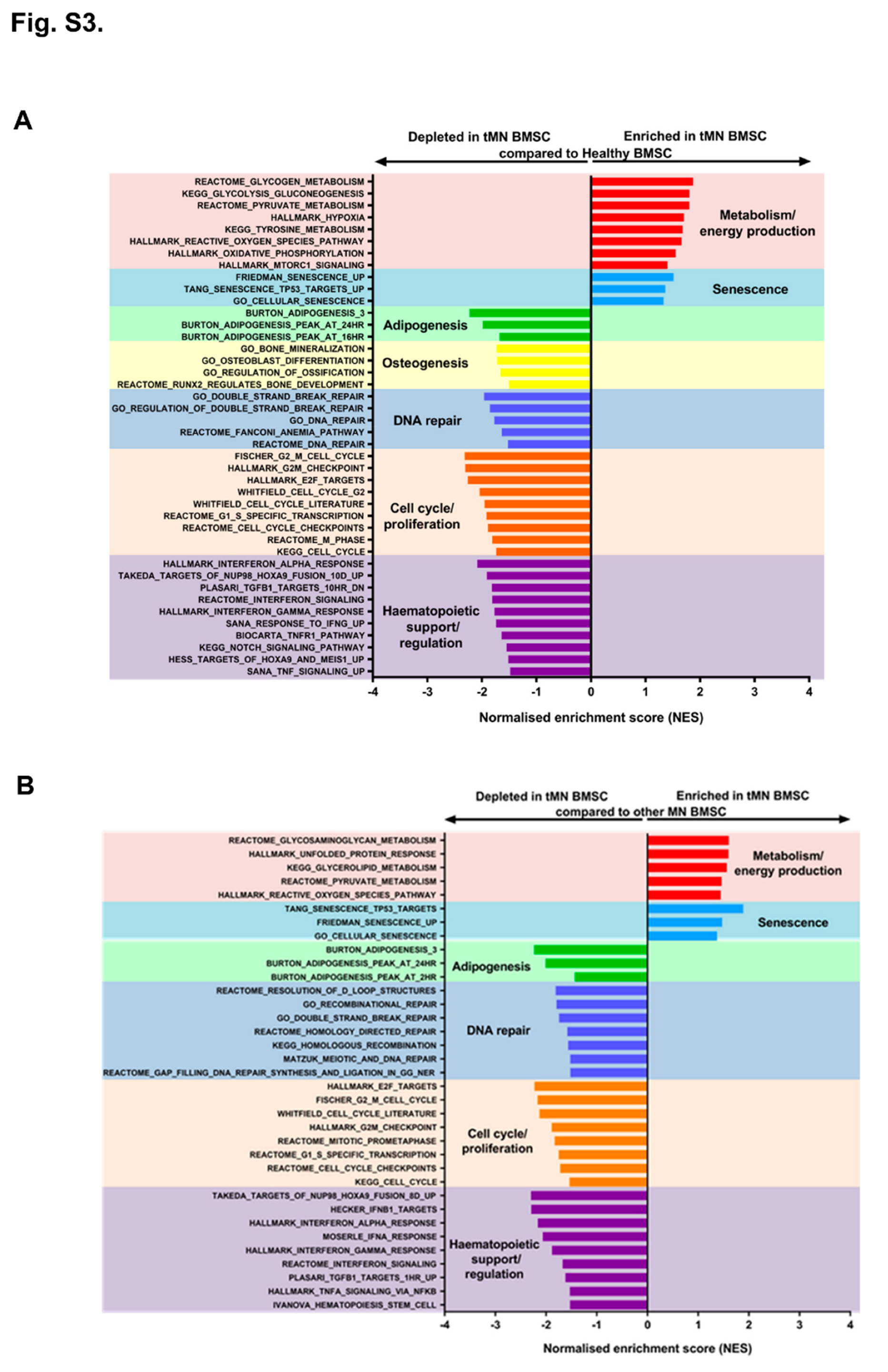
**

**Figure S3. Transcriptomic analysis of tMN BMSC showed enrichment of pathways involved in cellular senescence and metabolism while defective DNA damage repair and adipogenesis pathway compared to (A) Healthy and (B) other MN BMSC.** Gene set enrichment profiling of transcriptomic sequencing data. Red bars represent metabolism/ energy production-related data set. Light blue represents cellular senescence related signatures. Regulators of cellular senescence such as *CDKN1A,* *TNFRSF10D* gene expression levels were upregulated, while *FOS, IFITM1,* and *UHRF2* were significantly downregulated in tMN BMSC compared to Healthy controls (FDR < 0.1*, P* < 0.05*).*  Green bars represent adipogenesis- related signatures. Genes involved in adipogenesis such as *PNPLA2, TXNRD1, LGRF4, FGF2, FOS* and *TNFRSF19,* were highly deregulated in tMN compared to Healthy BMSC. Yellow represent osteogenesis –related data set. Dark blue bars represent DNA repair – related data set. Orange represents cell cycle/proliferation- related signatures. Purple bars represent hematopoietic support/regulation-related data set. NES indicates normalized enrichment score, positive score indicates positive enrichment in samples from tMN BMSC, and negative score indicates depletion in tMN BMSC; (B) comparison of transcriptome analysis of tMN with other, typical MN BMSC. Gene set enrichment profiling of transcriptomic sequencing data. Red bars represent metabolism/ energy production-related data set. Expression of genes regulating glycolysis such as *ME1, GPC1, STC2, PRPS1, PNPLA2* and *PLPP1* were highly deregulated in tMN compared to other MN BMSC (FDR < 0.1, *P* < 0.05). Light blue represents cellular senescence related signatures. Regulators of cellular senescence such as *CDKN1A,* *TNFRSF10D* gene expression levels were upregulated, while *FOS, IFITM1, PCNA,* and *UHRF2* were significantly downregulated in tMN BMSC compared to other MN (FDR < 0.1*, P* < 0.05*).*  Green bars represent adipogenesis- related signatures. Genes involved in adipogenesis such as *PNPLA2, CD36, TXNRD1, FOS, SLC44A2, ME1, LDLRAP1, SRDSA1, PTGDS, MTMR4* and *ARSB* were highly deregulated in tMN compared to other MN BMSC. Dark blue bars represent DNA repair – related data set. Orange represents cell cycle/proliferation- related signatures. Purple bars represent hematopoietic support/regulation-related data set. NES indicates normalized enrichment score, positive score indicates positive enrichment in samples from tMN BMSC, and negative score indicates depletion in tMN BMSC.


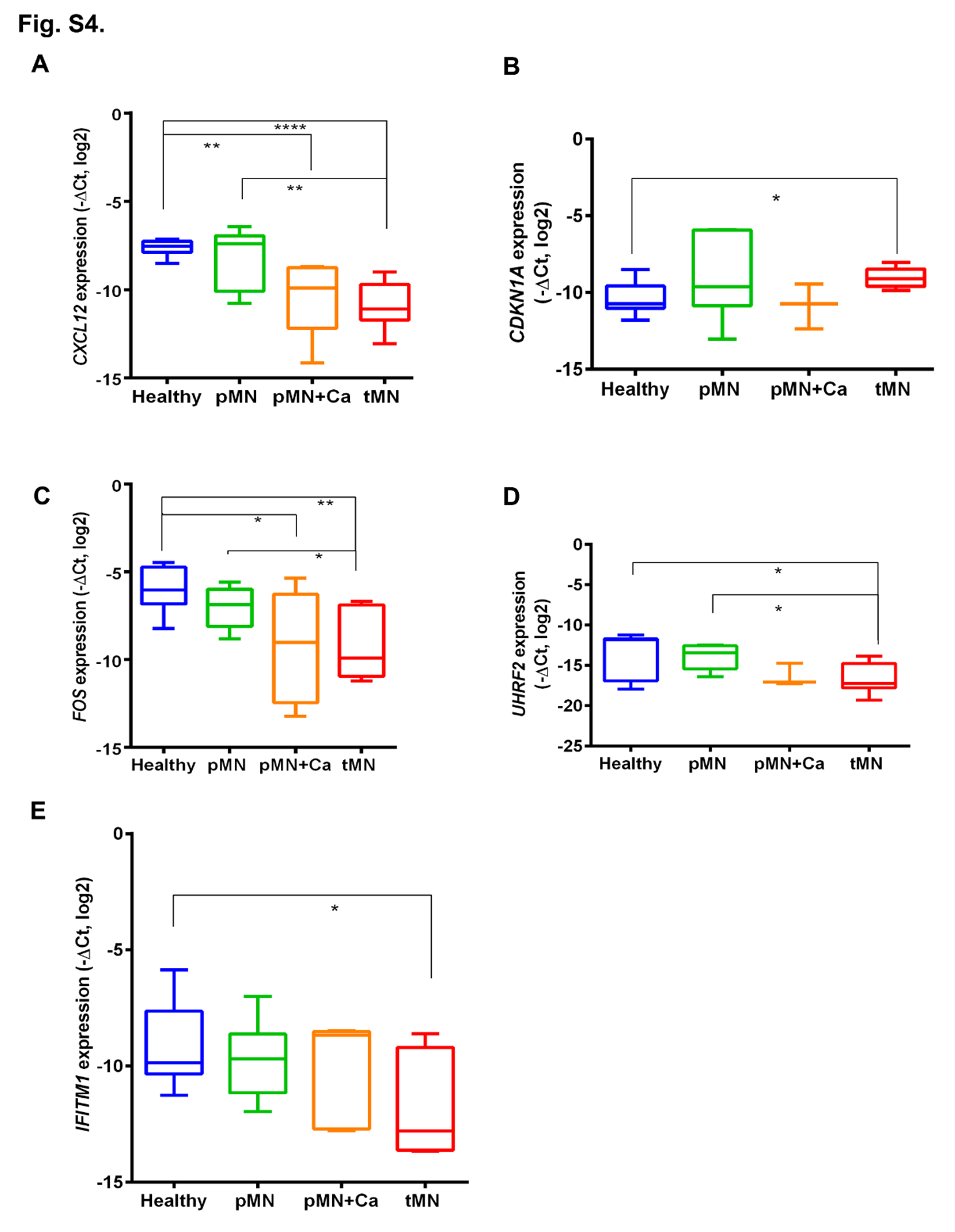


**Figure S4. Validation of differentially expressed genes in an independent cohort**: Expression of *CXCL12* (A), *CDKN1A* (B), *FOS* (C), *UHRF2* (D) and *IFITM1*(E) genes in Healthy (n = 7), pMN (n = 6), pMN+Ca (n = 5) and tMN (n = 7) BMSC. Boxplot shows the gene expression across 4 defined groups by qRT-PCR. Gene expression was determined relatively to *GAPDH* mRNA expression (ΔCt) and represented as –ΔCt in log2 scale. In boxplot, the horizontal line indicates the median, boxes indicate the interquartile range, and whiskers extend from the hinge to the smallest/largest value, at most 1.5 × interquartile range from the hinge. Unpaired Student’s t-test was used to detect statistically significant differences between cohorts. Asterisks display *P*-values **P* < 0.05, ***P* < 0.01, *P***** < 0.001.


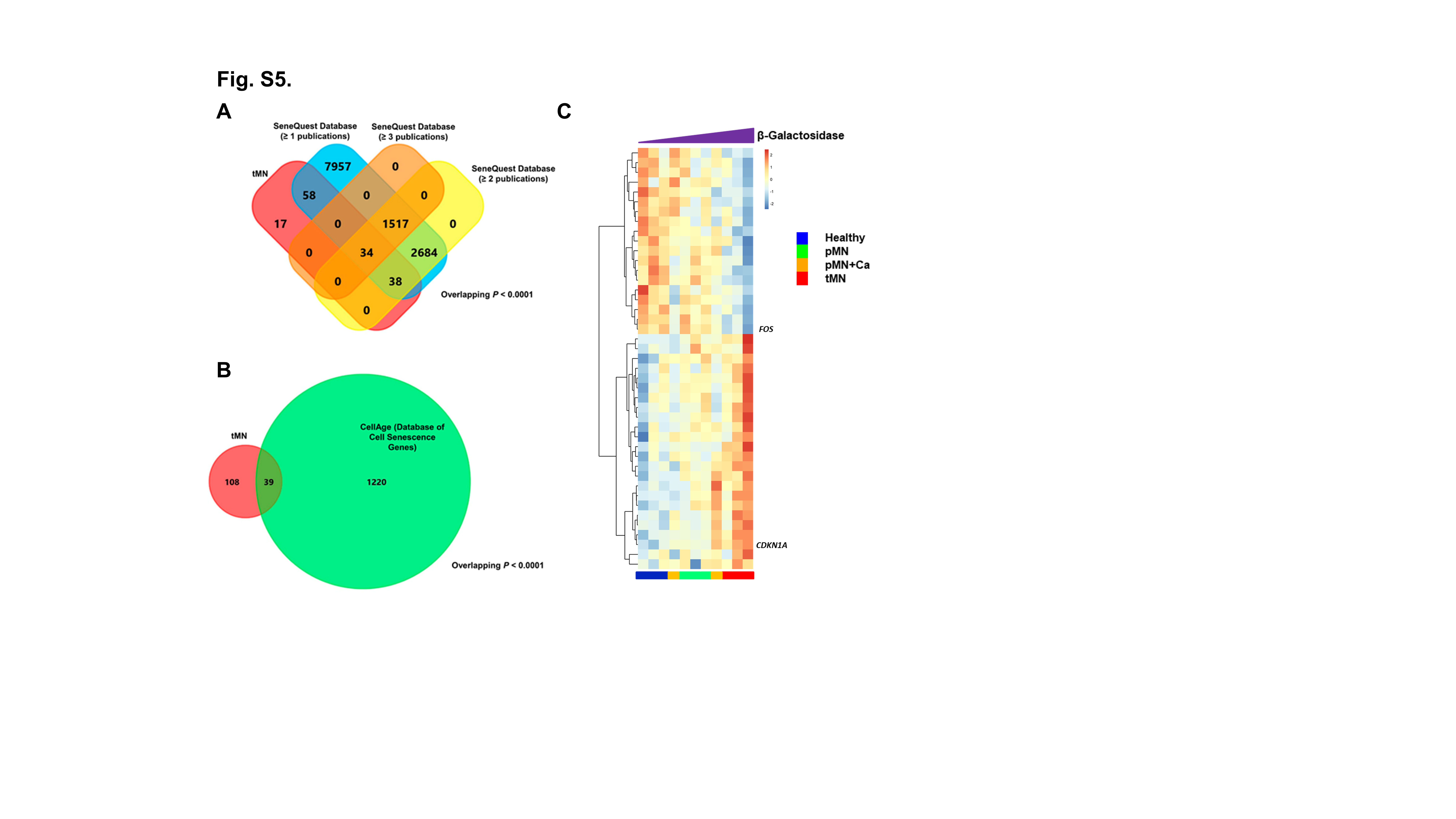


**Figure S5. Substantial number of the 147 genes differentially expressed between tMN and other MN BMSC were associated with cellular senescence in (A)** SeneQuest database ≥1, ≥2 and 3 publications and (B) CellAge database; (C) heatmap correlating β-Galactosidase levels and expression of senescence-associated genes in Healthy controls and MN. The differential gene expression was analyzed based on the β-Galactosidase value. Only gene with FDR P < 0.05 derived from edgeR analysis was considered as statistically significant.

**
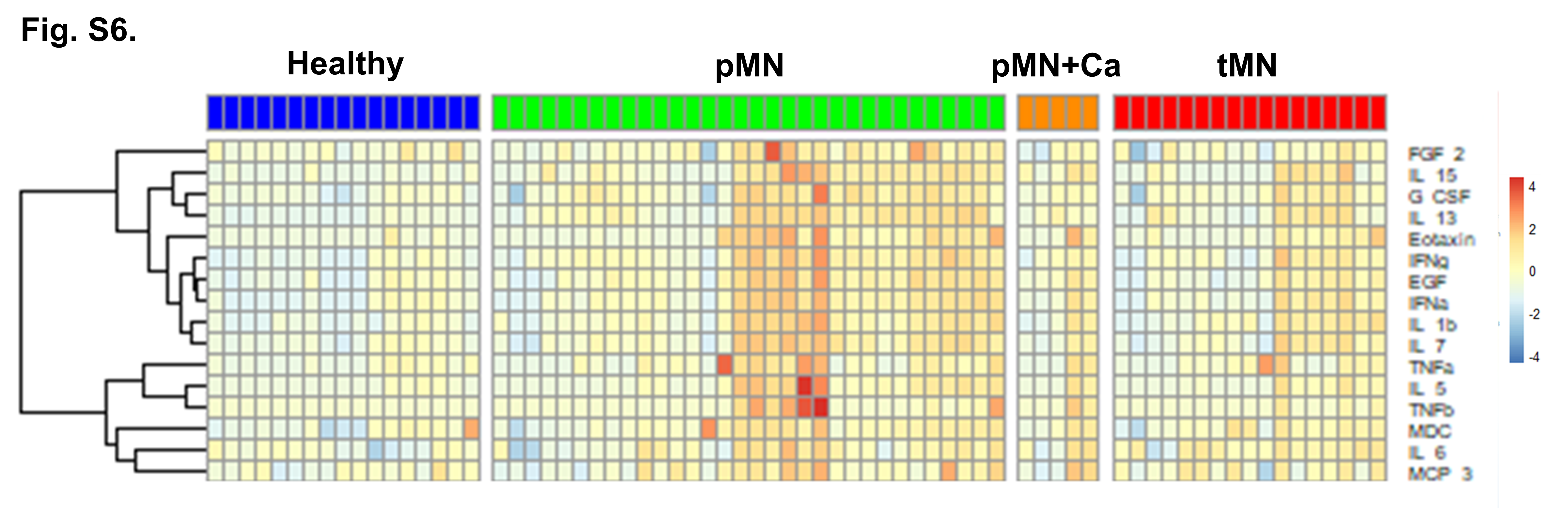
**

**Figure S6. Excessive secretion of multiple SASP-associated cytokines in tMN and other MN compared to Healthy BMSC condition media.** Hierarchical cluster analysis of Log-transformed cytokines levels of four cohorts (Healthy, n = 17; pMN, n = 32; pMN+Ca, n = 5; tMN, n = 17) using Ward’s method. Color scales represent cytokines levels (red indicates high levels; blue, low levels). The dendrogram on the left side of the heatmap reflects the order of cytokines.

**
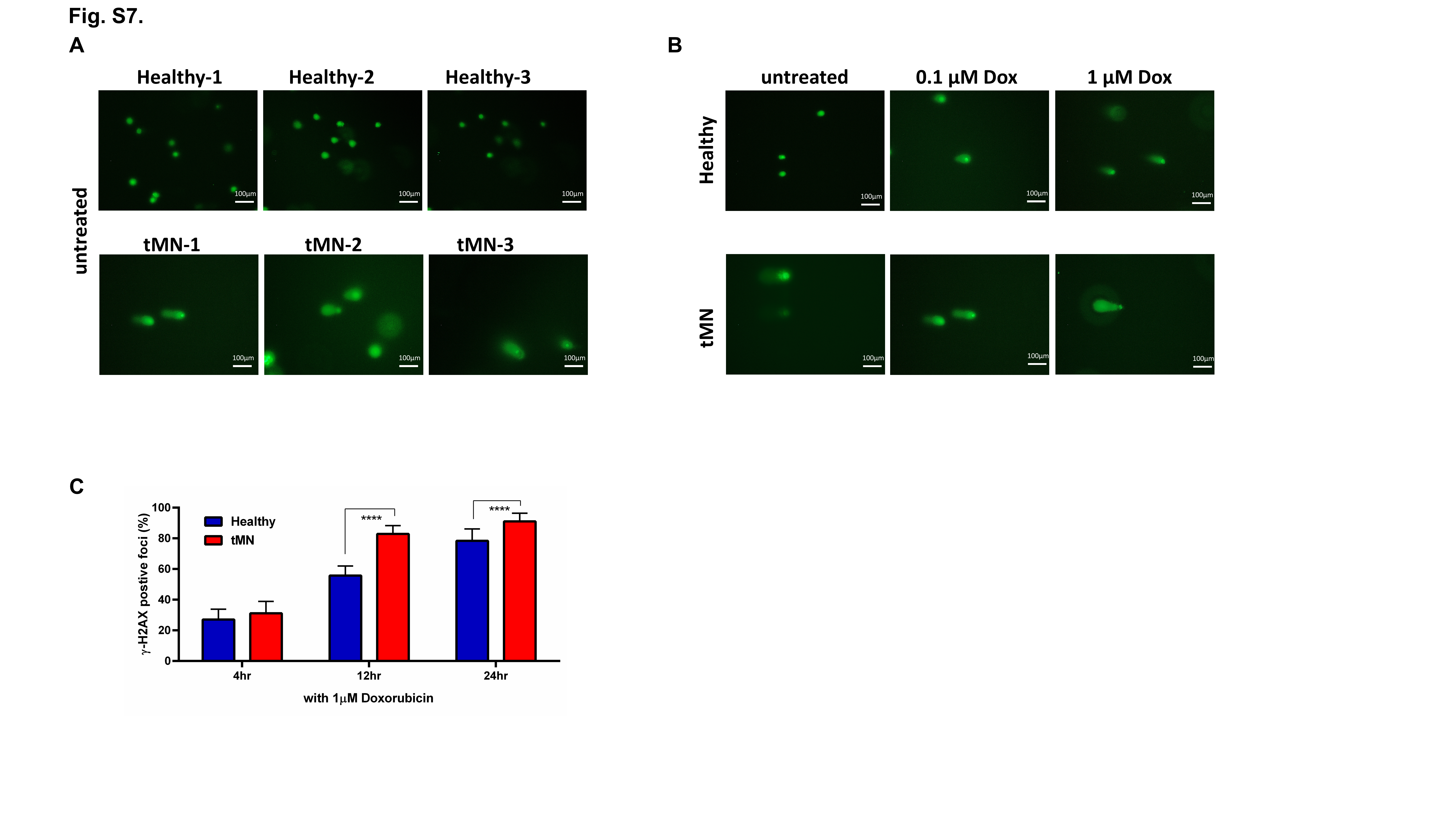
**

**Figure S7. tMN stromal cells are more sensitive to widely used DNA damaging chemotherapeutic drug, Doxorubicin.** (A) Alkaline comet assay comparing baseline DNA damage in (A) Health (n = 3) and (B) tMN BMSC (n = 3). Moreover, tMN BMSC are prone to DNA damage assessed by (B) alkaline comet assay (Doxorubicin 0.1µM and 1µM), and (C) γH2AX. γH2AX foci were significantly higher after 12 and 24 hours of exposure to 1µM Doxorubicin. All bars indicate mean, and all error bars indicate SD. Mann-Whitney test was used to detect statistically significant differences between cohorts. Asterisks display *P*-values **** *P* < 0.0001.

**
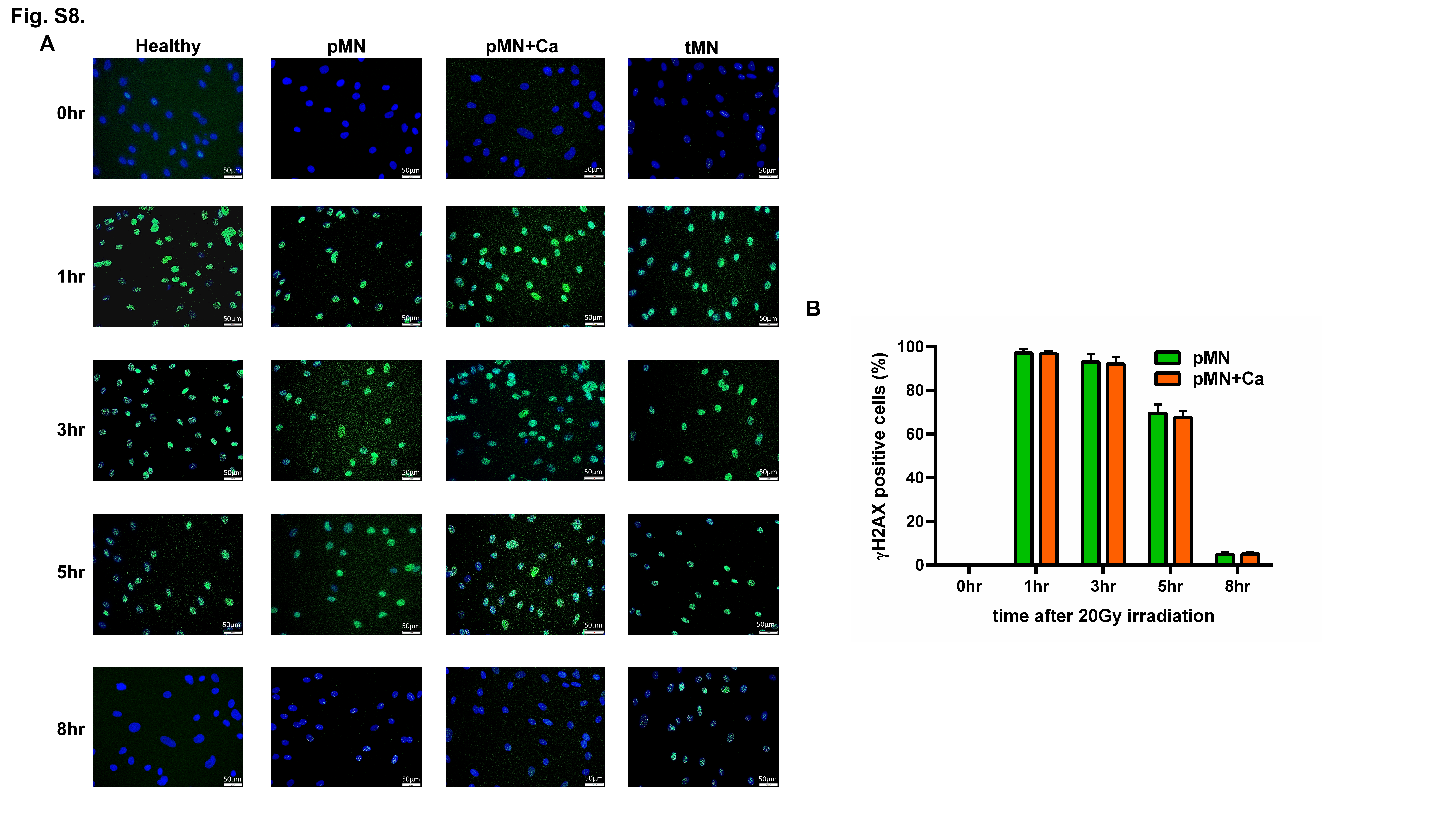
**

**Figure S8. Defective DNA damage repair potential of tMN compared to Healthy and other MN BMSC BMSC.** (A) Representative γH2AX staining and DAPI stained nuclei at 0, 1, 3, 5 and 8 hours post irradiation in pMN (n = 4), pMN + Ca (n = 4), tMN (n = 4) and Healthy (n = 4)-derived BMSC. Scale bars indicate 50μm. (B) Percentage of γH2AX positive cells at 0, 1, 3, 5 and 8 hours post irradiation in pMN and pMN+Ca. All bars indicate mean and all error bars indicate SD. Mann-Whitney test was used to detect statistically significant differences between cohorts.

**
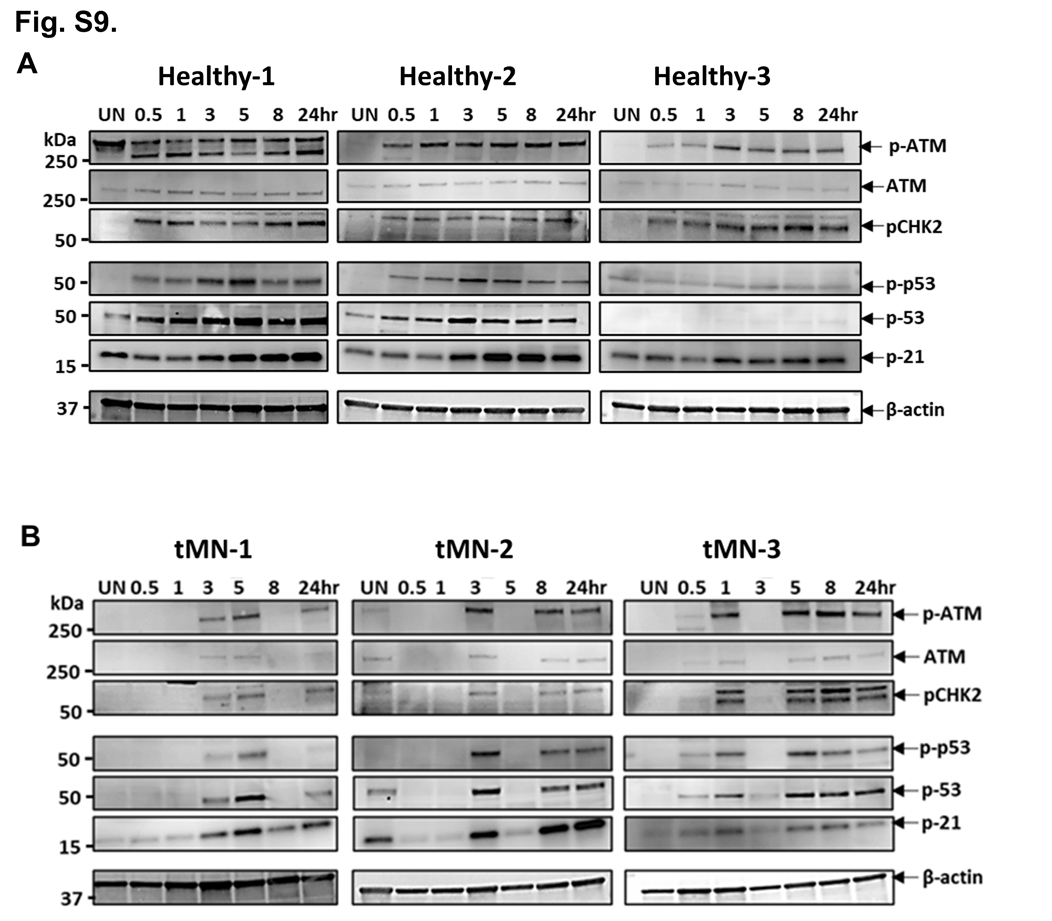
**

**Figure S9. DNA damage response in tMN and Healthy BMSC following sublethal irradiation**. Western blot analysis to assess DNA damage repair proteins ATM, CHK2, p53 and p21 prior to and at 0.5, 1, 3, 5, 8 and 24 hours post irradiation (20Gy) of (A) Healthy BMSC and (B) tMN BMSC.


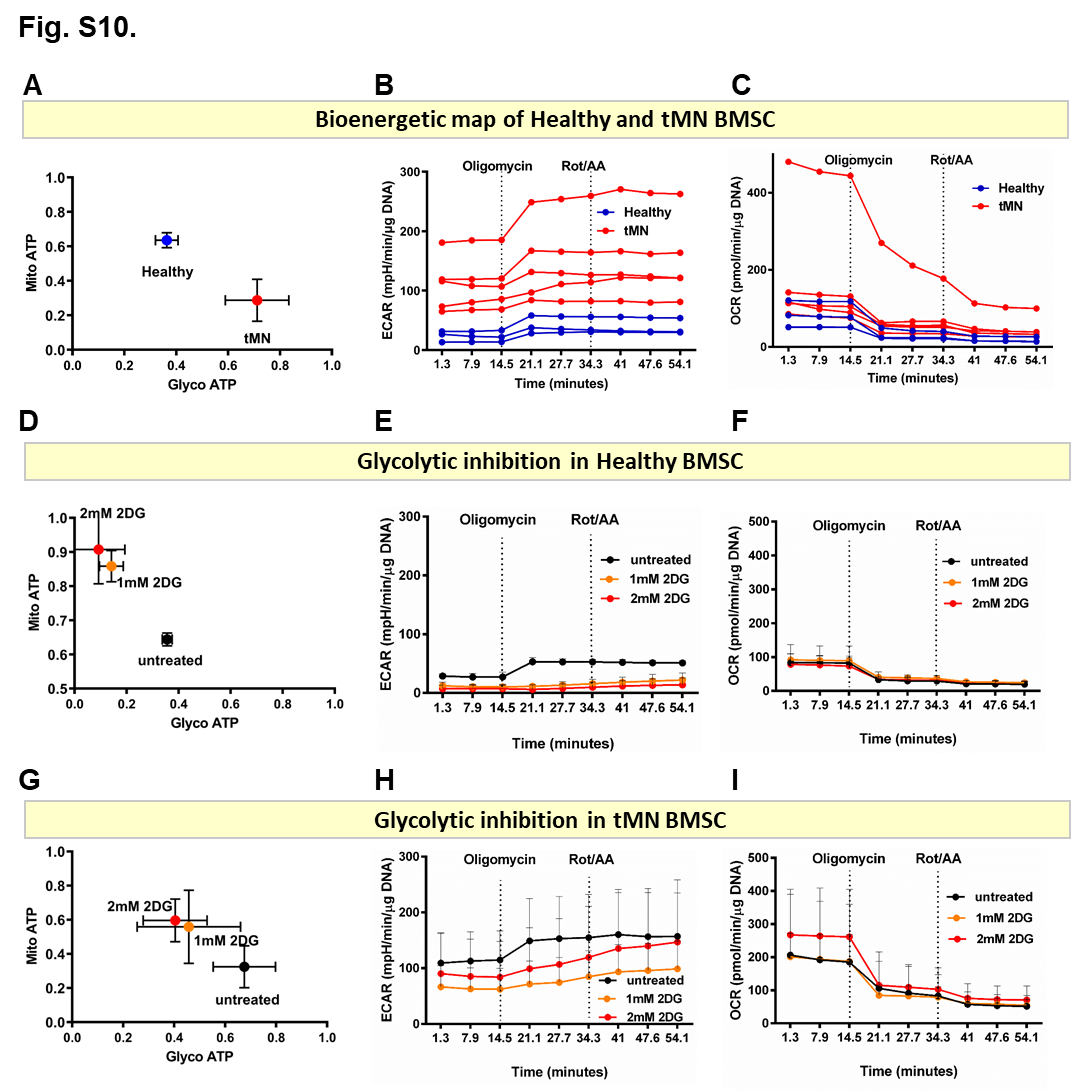


**Figure S10. Blockage of glycolytic pathway in tMN and Healthy BMSC switched bioenergetic metabolism to mitochondria without influencing oxygen consumption rate (OCR). (A)** energetic map of Healthy and tMN BMSC comparing mitochondrial ATP (mitoATP) vs. glycolytic ATP (glycoATP) production; (B) extracellular acidification rate (ECAR); (C) OCR measurements over time (minutes) in individual BMSC samples from Healthy and tMN. Effect of glycolysis inhibitor 2DG on bioenergetic production in Healthy BMSC assessed by (D) MitoATP vs. glycoATP energy production, and (E) ECAR and (F) OCR measurements of untreated and treated (1mM and 2mM 2DG) Healthy BMSC (n = 3). Effect of glycolysis inhibitor 2DG on tMN BMSC by assessing (G) mitoATP vs. glycoATP, (H) ECAR and (I) OCR measurements over time (minutes) between untreated and treated (1mM and 2mM 2DG) tMN BMSC (n = 3).


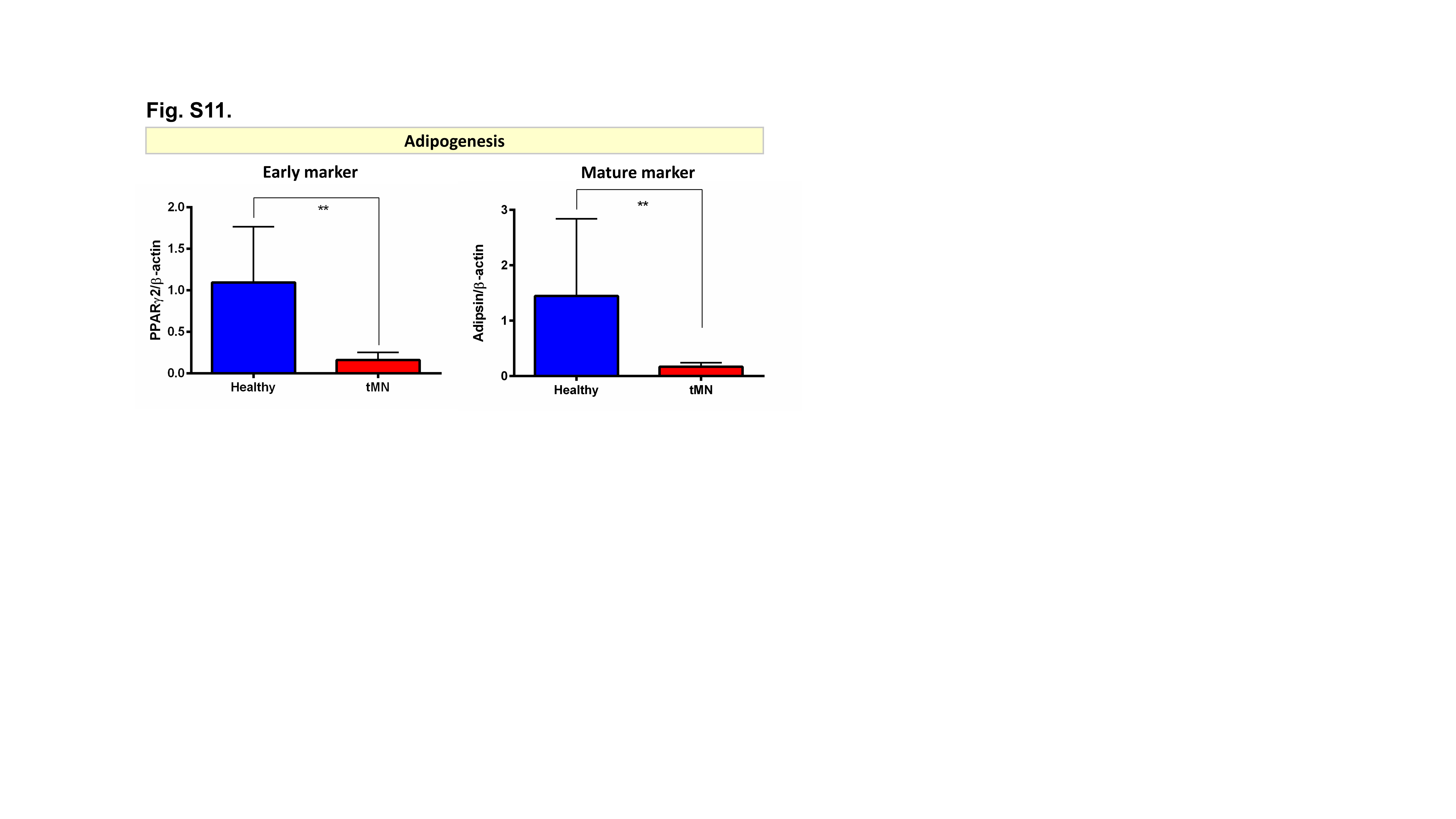


**Figure S11. Downregulation of genes associated with adipogenesis in tMN compared to Healthy BMSC.** All bars indicate mean and all error bars indicate SD. Mann-Whitney test was used to detect statistically significant differences between cohorts. Asterisks display *P*-values ** *P* < 0.01.


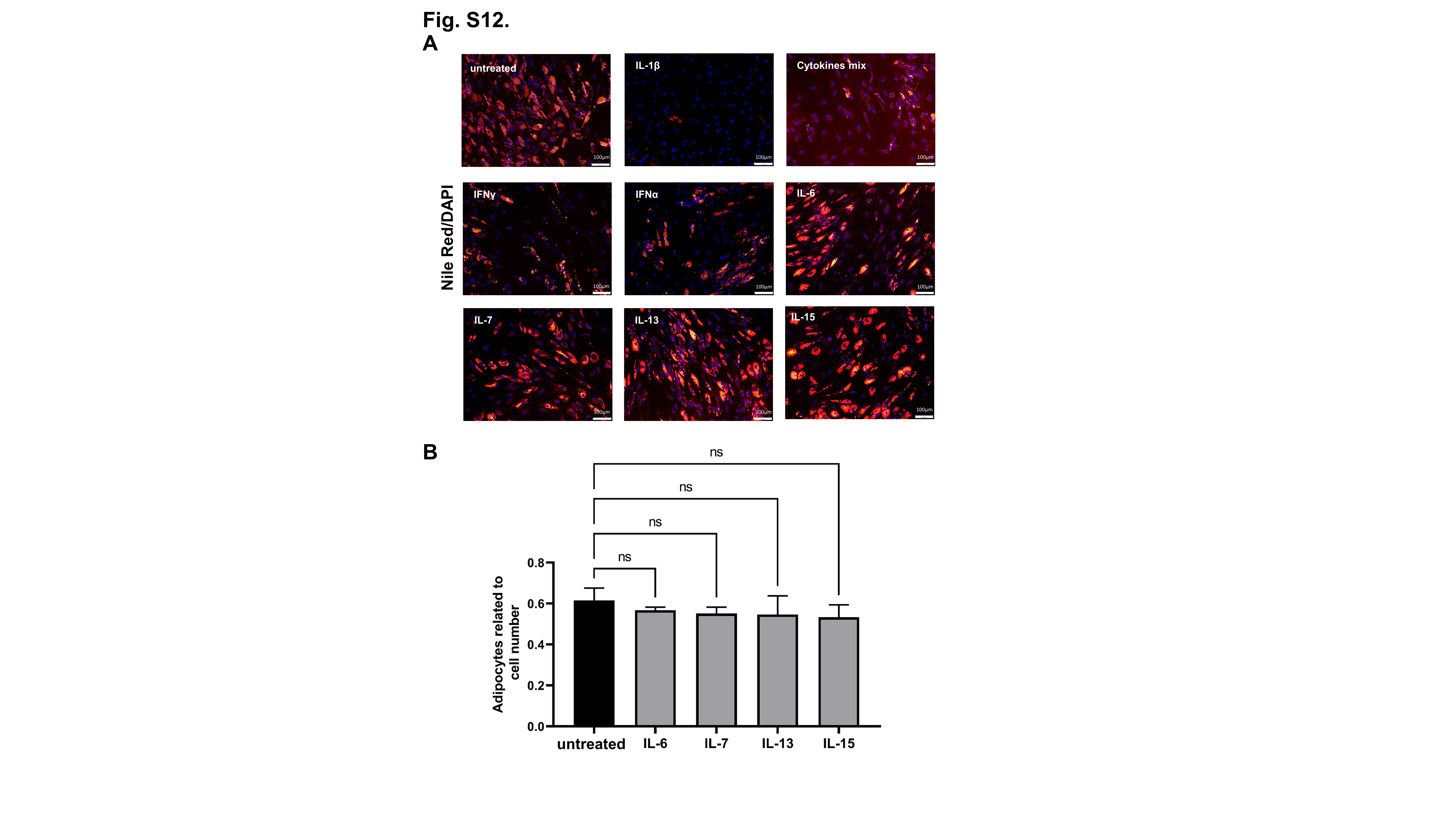


**Figure S12. *In vitro* culture with IL-6, IL-7, IL-13 and IL-15 did not block adipogenesis in Healthy BMSC.** (A) Representative micrographs of Healthy BMSC after exposure to the respective cytokines with scale bars indicating 100µm; (B) Quantification of Nile Red- labelled cells. All bars indicate mean and all error bars indicate SD. Mann-Whitney test was used to detect statistically significant differences between cohorts.

**
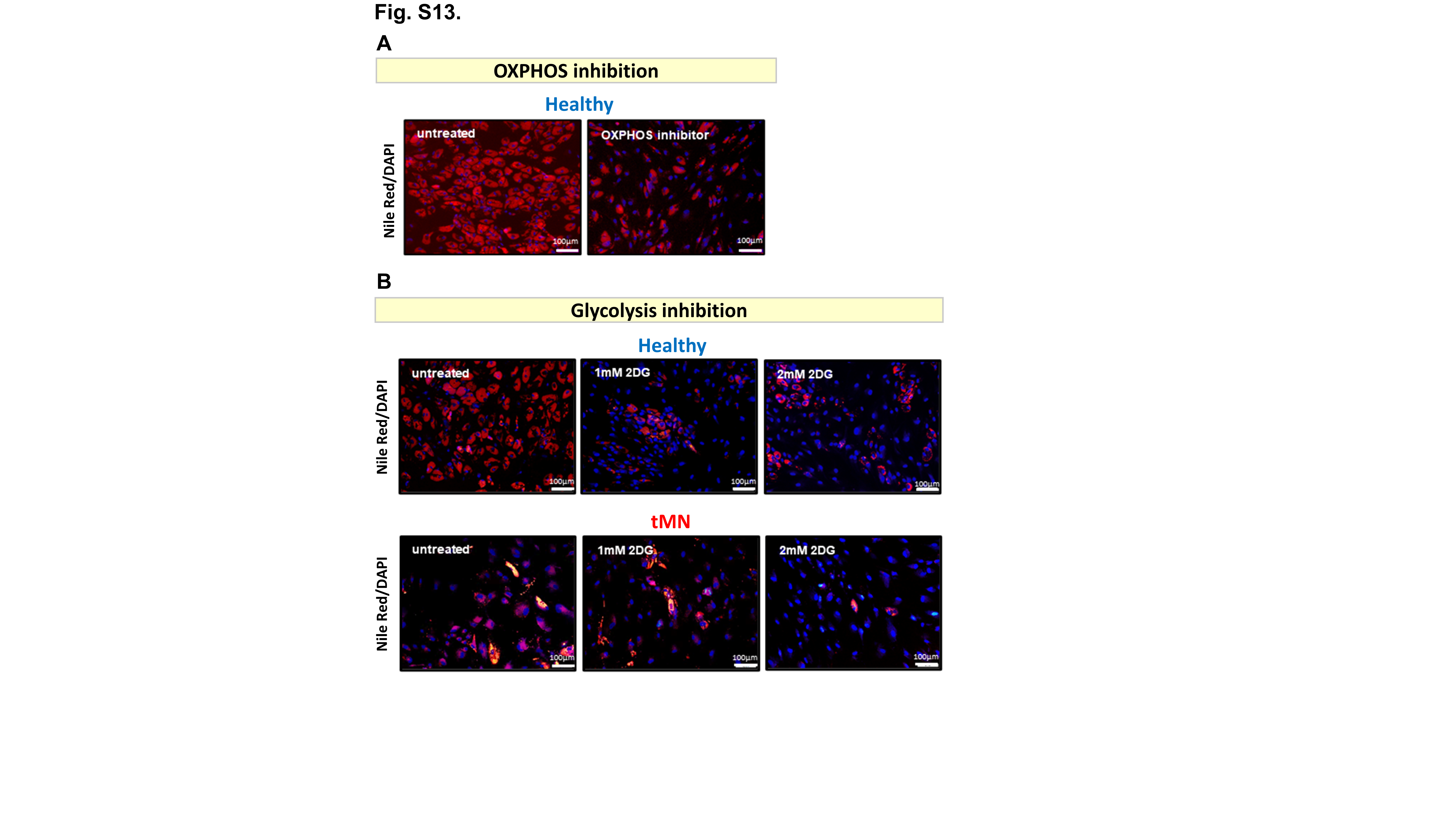
**

**Figure S13. Inhibition of glycolysis does not restore defective adipogenesis in tMN BMSC.** (A) Healthy BMSC were cultured in adipogenesis-induction media with or without OXPHOS inhibitor (IACS-010759) for 14 days followed by seven days without the inhibitor and adipogenesis was assessed after total of 21 days culture. Representative micrographs of Nile Red stained adipocytes; (B) Healthy BMSC (n = 3) and tMN BMSC (n = 3) were cultured under adipogenic induction conditions for 4 weeks either with or without 1mM and 2mM of 2DG, a glycolysis inhibitor, and number of adipocytes were evaluated by counting Nile Red-labelled cells and DAPI-labelled cell nuclei. Representative micrographs of Nile Red-stained cells and DAPI overlay in Healthy BMSC and tMN BMSC.

**
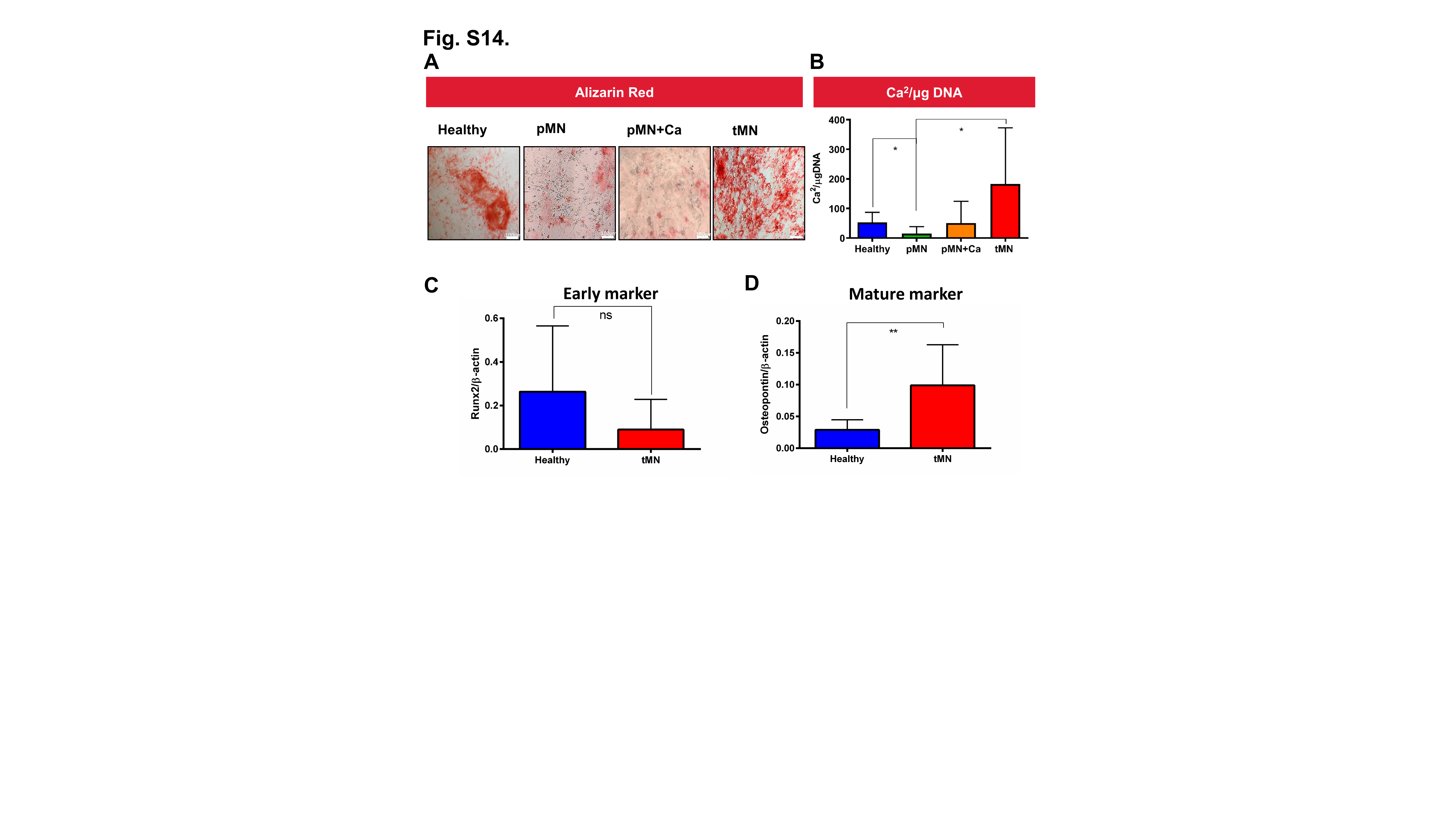
**

**Figure S14. Impaired osteogenic differentiation potential of tMN BMSC.** BMSC were cultured under osteogenic inductive conditions for 4 weeks (Healthy n = 8; pMN n = 5; pMN+Ca n = 3; tMN n = 11). (A) Representative photos of mineral deposits stained with Alizarin red; (B) Quantification of mineral content by measuring the concentration of Ca^2^ in an acid-solubilized matrix and normalizing them to the total DNA. mRNA expression of (C) Runx2 and (D) Osteopontin between Healthy and tMN BMSC. All bars indicate mean, and all error bars indicate SD. Scale bars indicate 100μm. Mann-Whitney test was used to detect statistically significant differences between cohorts. Asterisks display *P*-values **P* < 0.05, ***P* < 0.01.

**
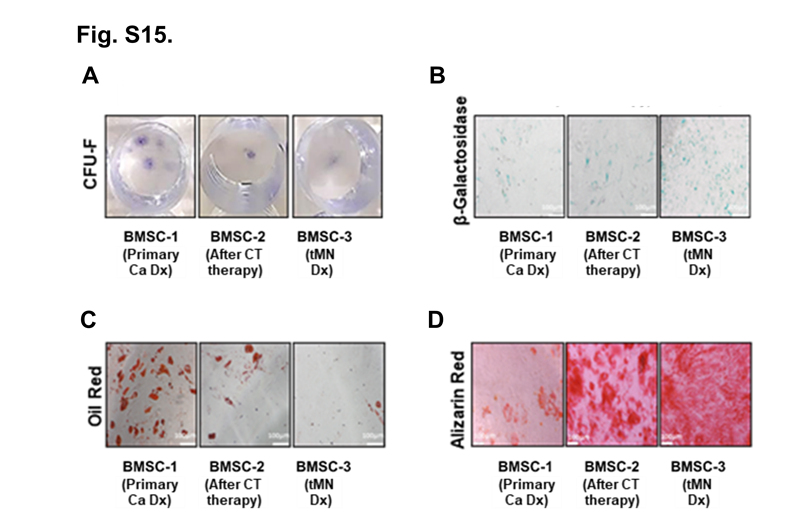
**

**Figure S15.** **Irreversible long-term BMSC damage is induced by CT in tMN patients.** (A) Clonogenic potential assessed by CFU-F. Representative micrographs of CFU-F colonies; (B) Senescence levels assessed by β-Galactosidase-stained cells. Representative micrographs: (C) adipogenic differentiation of BMSC visualized by Oil red staining; (D) The mineralization visualized by Alizarin Red staining at 21 days.
